# Selectivity of Per- and Polyfluoroalkyl Substance Sensors and Sorbents in Water

**DOI:** 10.1021/acsami.1c16517

**Published:** 2021-12-15

**Authors:** Yuqin Wang, Seth B. Darling, Junhong Chen

**Affiliations:** † Chemical Sciences and Engineering Division and Center for Molecular Engineering, Argonne National Laboratory, Lemont, Illinois 60439, United States; ‡ Advanced Materials for Energy-Water Systems Energy Frontier Research Center, Argonne National Laboratory, Lemont, Illinois 60439, United States; § Pritzker School of Molecular Engineering, University of Chicago, Chicago, Illinois 60637, United States

**Keywords:** PFAS, detection, adsorption, selectivity, water pollution, water treatment

## Abstract

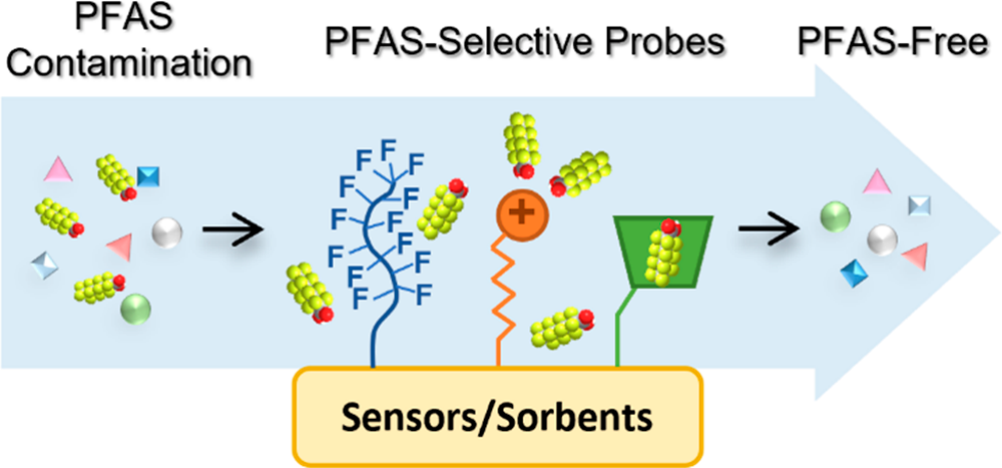

Per- and polyfluoroalkyl substances (PFAS) are a large group of engineered chemicals that have been widely used in industrial production. PFAS have drawn increasing attention due to their frequent occurrence in the aquatic environment and their toxicity to animals and humans. Developing effective and efficient detection and remediation methods for PFAS in aquatic systems is critical to mitigate ongoing exposure and promote water reuse. Adsorption-based removal is the most common method for PFAS remediation since it avoids hazardous byproducts; in situ sensing technology is a promising approach for PFAS monitoring due to its fast response, easy operation, and portability. This review summarizes current materials and devices that have been demonstrated for PFAS adsorption and sensing. Selectivity, the key factor underlying both sensor and sorbent performance, is discussed by exploring the interactions between PFAS and various probes. Examples of selective probes will be presented and classified by fluorinated groups, cationic groups, and cavitary groups, and their synergistic effects will also be analyzed. This review aims to provide guidance and implication for future material design toward more selective and effective PFAS sensors and sorbents.

## 1 Introduction

Over the past few years, per- and polyfluoroalkyl substances (PFAS) have rapidly attracted attention as an emerging threat to both our environment and human health. PFAS are a large group of engineered chemicals whose structures contain either fully or partially fluorinated carbon chains with different chain lengths and functional groups. According to a recent report, the number of PFAS molecules on the global market that have been assigned with a chemical abstracts service (CAS) registry number has exceeded 4700.^1^ Together with unidentified species, the total number of PFAS molecules may be as high as 5000–10000, and the number is still increasing.^2^ PFAS have been manufactured and used in many aspects of our lives since the 1940s. Products that contain PFAS include, but are not limited to, aqueous film-forming foam (AFFF), nonstick cookware, fast food wrappers, stain-resistant carpets and fabrics, cleaning products, and personal care products. During the production and application processes of these products, PFAS will be released into our environment, transported within and across different media (air, water, soil, and food), and finally taken up by aquatic organisms, plants, and humans.^2^ Once PFAS enter our environment or organisms, they resist degradation/metabolization due to the strong carbon–fluorine bonds (C–F, 105.4 kcal/mol).^3^ The persistence and accumulation of PFAS pose a major threat to the environment and our health. Exposure to PFAS might cause or contribute to many adverse health effects of organs such as the thyroid, kidney, and liver and may also lead to high cholesterol and even cancer.^4−9^

**Figure 1:**
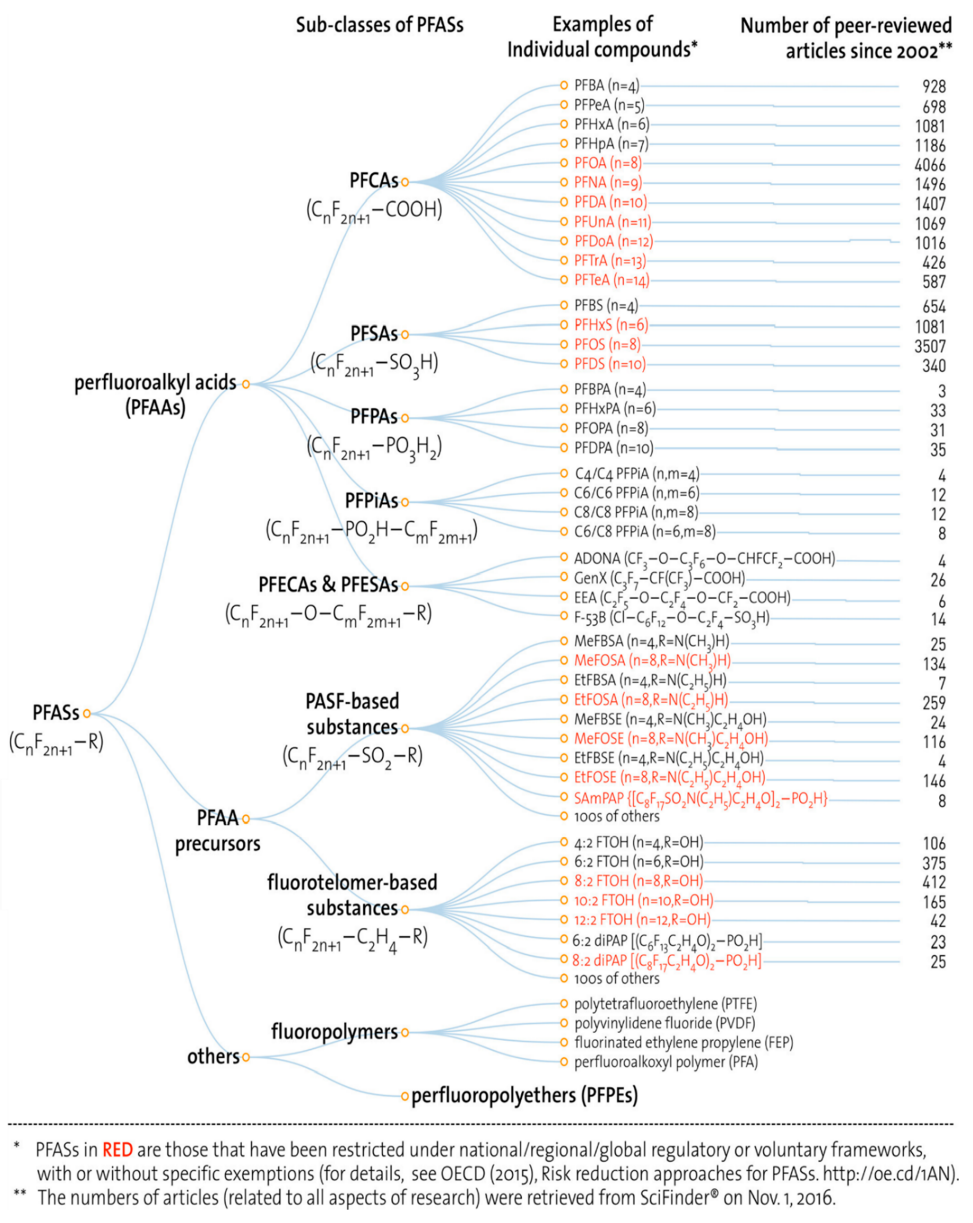
Classification of PFAS, including examples of individual PFAS and the number of peer-reviewed articles on them since 2002. Reprinted with permission from ref (10). Copyright 2017 American Chemical Society.

PFAS can be sorted into three categories according to a simple classification program: perfluoroalkyl acids (PFAAs), PFAA precursors, and others (fluoropolymers and perfluoropolyethers (PFPEs)) (Figure 1).^10^ PFAAs have the most toxicological information,^11^ among which perfluorooctanoic acid (PFOA) and perfluorooctanesulfonic acid (PFOS) are the most common species found in the environment and also the most heavily studied. These substances used to be extensively manufactured globally but were voluntarily and gradually phased out in the United States since 2000 due to ecological and health concerns.^12,13^ However, PFOA and PFOS are still present in our environment due to their persistence and imports from other countries.^14^ Chemical manufacturers in the United States are seeking alternative PFAS molecules to replace legacy long-chain species. Replacement chemicals include short-chain homologues of the long-chain species like perfluorobutanesulfonic acid (PFBS), fluorotelomer-based products including 6:2 fluorotelomer-based compounds in AFFF formulations, and per- and polyfluoroalkyl ether substances such as the ammonium salt of 2,3,3,3-tetrafluoro-2-(1,1,2,2,3,3,3-heptafluoropropoxy)propanoic acid (GenX).^2^ PFAAs can be further divided into long-chain PFAS and short-chain PFAS based on the carbon chain length or into perfluoroalkyl sulfonic acids (PFSAs) and perfluoroalkyl carboxylic acids (PFCAs) based on the type of functional groups.^15,16^ PFAA precursors can be converted to PFAAs through various transformation pathways.^2^ While fluoropolymer and PFPE may not be degraded into PFAAs, they may release PFAAs in their final products, industrial waste, or during incineration.^11^

**Figure 2:**
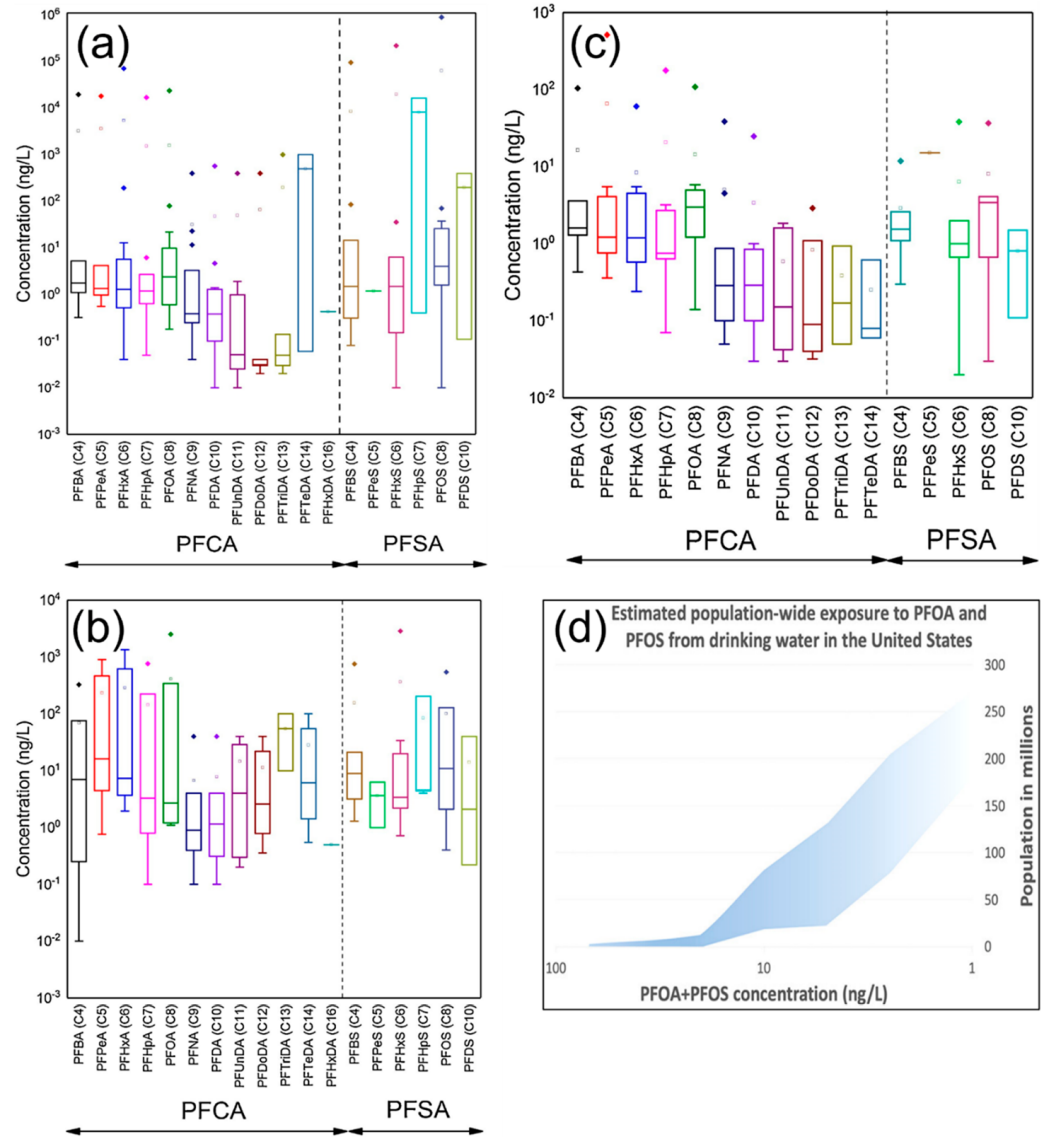
PFAS occurrence and exposure. Concentrations of selected PFAS in (a) surface water, (b) groundwater, and (c) drinking water. The data are analyzed according to the concentration range of selected PFAS compounds in various countries. Reprinted with permission from ref (18). Copyright 2020 Elsevier. (d) Population-wide exposure to PFOA and PFOS from drinking water in the United States. Reprinted with permission from ref (22). Copyright 2020 American Chemical Society.

PFAS are widely detected in aquatic systems including wastewater, surface water, groundwater, and drinking water. Their concentrations range from several pg/L to μg/L, among which several ng/L is the most common case.^17,18^Figure 2a–2c shows the occurrence of different PFAS in various water matrices (surface water, groundwater, and drinking water) summarized by Phong Vo et al. in 2020. These data were analyzed based on the concentration range of specific PFAS molecules in various countries including the United States, Canada, France, China, Australia, Sweden, Vietnam, Finland, Uganda, Malta, and South Korea.^18^ In 2016, the United States Environmental Protection Agency (USEPA) released a nonenforceable health advisory level of 70 ng/L (70 ppt) for lifetime exposure of PFOA and PFOS.^19^ As of May 2020, nine U.S. states have developed more stringent drinking water standards or guidelines for PFOA and PFOS, ranging from 8 to 40 ng/L.^20^ In September 2020, the European Food Safety Administration (EFSA) established a group tolerable weekly intake (TWI) of 4.4 ng/kg of body weight per week for the sum of perfluorohexanesulfonic acid (PFHxS), PFOA, PFOS, and perfluorononanoic acid (PFNA).^21^ Food contamination by PFAS can also be related to PFAS-contaminated water sources, such as through the contaminated water used to grow the food and through PFAS in animals via feed and water. In 2020, Andrews et al. from the Environmental Working Group (EWG) analyzed publicly available data sources of PFAS occurrence in drinking water in the United States (Figure 2d). They estimated that over 200 million people may receive tap water with a combined PFOA and PFOS concentration at or above 1 ng/L; 18–80 million people may receive more than 10 ng/L; and 0.4–1 million people likely drink tap water having more than 70 ng/L of PFOA and PFOS.^22^ Facing the wide occurrence of PFAS in our water systems and their potential toxicity to humans and the environment, there is an urgent research need to develop effective and efficient methods to both detect PFAS concentrations and remove these substances from our aqueous systems. However, the complexity of the natural aqueous matrices and the relatively low concentration of PFAS in water compared to other coexisting species like inorganic ions^23^ and dissolved organic matter^24^ (whose concentrations are commonly from μg/L to mg/L) make the monitoring and remediation of PFAS difficult.

Standard detection methods for PFAS mainly rely on chromatography coupled with mass spectrometry in professional laboratories.^25^ The EPA announced official analytical methods (“Method 537” and “Method 533”) to analyze 29 different PFAS in drinking water, which utilize solid-phase extraction (SPE)-enabled liquid chromatography-tandem mass spectrometry (LC-MS/MS).^26,27^ This method provides sufficient accuracy and sensitivity, but it requires skilled personnel, sophisticated, and expensive instruments and tedious sample preparation and data collection processes. Recently, several efforts have been made to design new PFAS sensing devices that are not MS-based, aiming to lower the cost, testing time, and complexity of PFAS detection.^28^ Sensors that incorporate nanoscale materials have some inherent advantages, such as quick response, easy operation, low cost, or the potential to build hand-held devices to achieve on-site detection, and thus they represent promising candidates for future PFAS detection. However, most of these sensors currently lack either sufficient sensitivity^29−31^ or selectivity.^32−34^ The key to address these drawbacks is to improve the interactions between the sensor materials and PFAS targets.

For PFAS remediation, a variety of methods including adsorption, chemical/electrochemical oxidation, photolysis, biotransformation, incineration, and fractionation could be effective.^35^ Among them, adsorption is arguably the most feasible, mature, and environmentally friendly method.^35^ Commercially available sorbents including activated carbon (AC) and anion exchange resins (AERs) exhibit remarkable adsorption capacity,^36^ but they are not perfect sorbents, with drawbacks like rapid breakthrough and slow adsorption rate.^37^ Moreover, the selectivity of those sorbents is inadequate. To enhance the selectivity of PFAS adsorption, various synthetic sorbents have been produced by incorporating multiple functional groups and spatial structures that could exhibit specific interactions toward PFAS.^38−40^ Although the selectivity of sorbents has been taken into consideration in these synthetic sorbents, the mechanism behind the selectivity is not fully understood. Design principles of PFAS sorbents based on specific interactions between PFAS and material surfaces have not yet been established.

Published reviews focusing on PFAS detection including PFAS sensors^25,28,41,42^ and remediation including PFAS sorbents^36,43−46^ have provided valuable perspective on these topics. These reviews focused on the classification of sensor or sorbent materials, the working mechanisms behind sensors, and the evaluation of sensing or adsorption performance. There remains a need, however, for a thorough discussion on the selectivity of PFAS sensors or sorbents, which is a critical factor impacting the sensing and adsorption effectiveness. Interaction mechanisms between PFAS and sorbent materials,^43,44^ which are the basis to understand the selectivity, have been discussed at a material level but not on a molecular level, which would provide important insights into materials design for PFAS detection and remediation. The objective of this review is to provide an overview of the state-of-the-art technologies for PFAS sensing and adsorption in water matrices with a focus on material selectivity and to provide insights into how to choose probes for sorbents/sensors based on specific interactions between PFAS and functional groups (molecular level). PFAS sensors and sorbents are integrated in this review because the binding selectivity underlying those two types of functional materials is similar, allowing for knowledge sharing. To better understand the current stages of development for PFAS sensors and sorbents, we first summarize PFAS sensors based on the sensing mechanism and PFAS sorbents based on the type of materials. The selectivity of those sensors and sorbents is discussed together with an understanding of the interactions behind the selectivity. Examples of probes that sorbents and sensors utilize to achieve PFAS specificity will be presented and organized by functional groups. Finally, future directions for developing a more selective and effective PFAS sorbent and sensor will be discussed.

## 2 PFAS Sensors

**Figure 3:**
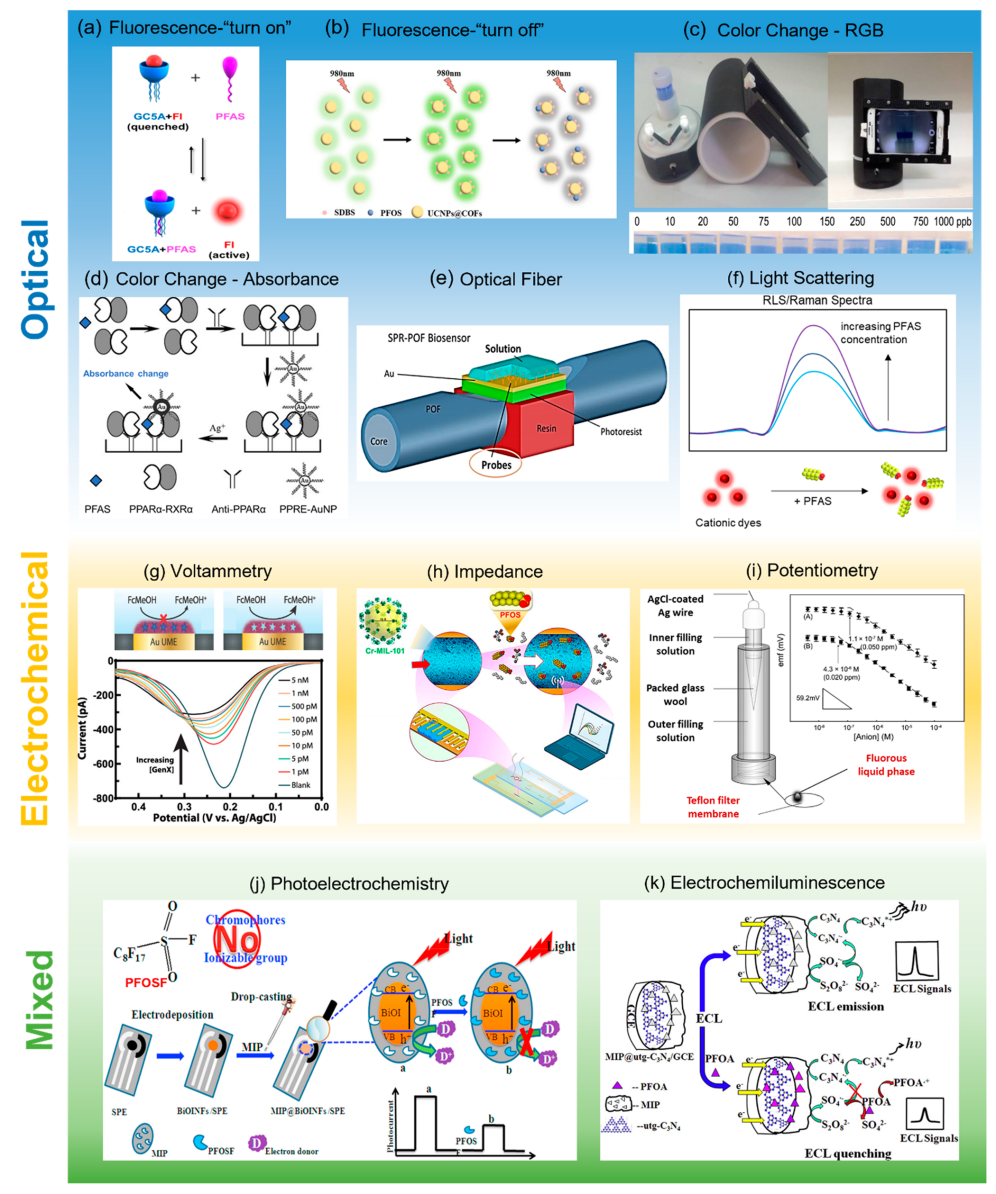
PFAS sensors can be classified into (a–f) optical sensors, (g–i) electrochemical sensors, and (j,k) mixed sensors according to their sensing mechanisms. (a) A “turn-on” fluorescent sensor using fluorescein (Fl) as the emissive species and guanidinocalix[5]arenes (GC5A) as the quencher and the PFAS-capturing probe. Reprinted with permission from ref (30). Copyright 2019 Springer Nature. (b) A “turn-off” fluorescent sensor using UCNPs@COF nanoparticles as the sensing probe for PFOS. Reprinted with permission from ref (50). Copyright 2019 American Chemical Society. Further permissions related to the material excerpted should be directed to the ACS. (c) A smartphone-app-based portable sensor based on the color change of dyes upon conjugation with PFOA. Reprinted with permission from ref (52). Copyright 2018 Elsevier. (d) A colorimetric sensor for PFAS based on the interaction between PPRE-modified gold nanoparticle probes and PPARα activated by PFAS. Adapted with permission from ref (54). Copyright 2011 Elsevier. (e) A SPR optical fiber biosensor using an ad hoc produced monospecific antibody as the PFAS-capturing probe. Reprinted with permission from ref (56). Copyright 2018 Elsevier. (f) Light scattering based PFAS sensor using cationic dyes as the probe. (g) A MIP-modified microelectrode for voltammetric detection of GenX with ultrasensitivity. Reprinted with permission from ref (61). Copyright 2019 American Chemical Society. Further permissions related to the material excerpted should be directed to the ACS. (h) A MOF-based microfluidic impedance sensor for PFOS. Reprinted with permission from ref (64). Copyright 2020 American Chemical Society. (i) ISEs with fluorous anion-exchange membranes for the potentiometric detection of PFAS. Inset shows the potentiometric response curves for (A) PFO^–^ and (B) PFOS^–^. Reprinted with permission from ref (65). Copyright 2013 American Chemical Society. (j) A disposable photoelectrochemical sensing strip for PFAS. Reprinted with permission from ref (67). Copyright 2018 Elsevier. (k) A ECL sensor for PFOA using molecularly imprinted ultrathin graphitic carbon nitride nanosheets as the sensing probe. Reprinted with permission from ref (69). Copyright 2015 Elsevier.

Although LC-MS/MS is currently the gold standard for PFAS detection with excellent sensitivity (lower detection limit ranges from 0.53 to 2.8 ng/L for 18 common PFAS molecules^26^), accuracy, and reliability, it cannot meet the growing need for a rapid, portable, low-cost, and user-friendly detection method. To overcome these drawbacks, a variety of PFAS sensors based on different devices and materials are being investigated. Emerging sensors tend to use relatively simple devices to achieve fast PFAS sensing with a lower cost, and some of them even have the potential for on-site use. Generally, current PFAS sensors can be classified into three categories according to their sensing mechanisms: optical sensors, electrochemical sensors, and mixed sensors (sensors that combine the optical and electrochemical mechanisms together in the sensing process) (Figure 3).

Optical sensors transform chemical information like concentration to measurable optical signals. There are multiple optical phenomena that could be utilized to produce meaningful signals, for example, fluorescence, color change, surface plasmon resonance (SPR), and light scattering (Figure 3a–f).^42^ Among these phenomena, fluorescence is the most utilized due to its versatility. Fluorescent sensors can be classified into “turn-on” or “turn-off” types, which correspond to enhanced or quenched fluorescence caused by PFAS conjugation, respectively.^42^ Usually, fluorescent sensors would have at least one fluorescent material (probe) that can emit fluorescence upon excitation. This fluorescent probe could interact with PFAS through various modes, such as electrostatic interaction or hydrophobic interaction, and then change its emissive properties, which leads to different degrees of changes in fluorescent signal depending on PFAS concentration. For “turn-on” types, the fluorescent probe is either originally weakly emissive (e.g., a molecularly imprinted chitosan-doped carbon quantum dot),^44^ nonemissive (e.g., aggregation-induced emission luminogens),^47^ or emissive but quenched by a paired quencher (e.g., fluorescein and guanidinocalix[5]arenes) (Figure 3a).^30^ After this fluorescent probe conjugates with PFAS molecules, the fluorescence emission is activated or strengthened, and the degree of this signal change can be correlated to PFAS concentrations. Similarly, “turn-off” fluorescence sensors exhibit decreasing fluorescent signal when the fluorescent probe interacts with PFAS molecules, and the extent of the fluorescence reduction can be converted to PFAS concentrations. Carbon quantum dots,^48^ cationic porphyrin,^49^ and upconversion nanoparticles (UCNPs)^50^ have been utilized as the fluorescent probe for this type of sensor. While most optical sensors have a limit of detection (LOD) around the ppb or ppm level,^42^ one “turn-off” fluorescent sensor using UCNPs as the fluorescent probe showed superior sensitivity among all PFAS detection methods, with a LOD of 75 pg/L (0.15 pM) for PFOS, which is much lower than USEPA’s advisory level (70 ng/L).^50^ In this work, the fluorescent UCNPs were functionalized with covalent–organic frameworks (COFs) to form composite UCNPs@COFs (Figure 3b). The COF layer on the surface of UCNPs could not only capture and enrich PFOS onto the surface but also improve the fluorescence quantum yield. The fluorescence response of UCNPs@COFs to PFOS could also be significantly enhanced by anion surfactants like sodium dodecyl benzenesulfonate (SDBS). However, due to the poor dispersion and weak emission of UCNPs@COFs in water, all the sensing tests were performed in the dimethylformamide (DMF) matrix, which required extra sample pretreatment.

Color change is another important mechanism for PFAS optical sensors, and this signal change can be measured by either an absorbance spectrum^51^ or direct color reading based on RGB values (Figure 3c).^52^ Color change could be induced by reactions of chromogenic species,^53^ aggregation of gold nanoparticles (AuNPs),^51^ or conjugation with dyes^52^ upon mixing PFAS molecules with the sensor. One sensor that incorporated AuNPs into a bioassay achieved excellent sensitivity, with a LOD of 5 ng/L (10 pM).^54^ This sensor is designed based on the fact that PFOS is a special agonist for peroxisome proliferator-activated receptor α (PPARα). The AuNPs were functionalized with peroxisome proliferator-response elements (PPREs), which could conjugate with PPARα only when the latter was activated by its ligands (e.g., PFOS). Silver was added to the system to amplify the optical density. According to this principle, PFOS concentrations could be quantified by measuring the absorbance intensity caused by the silver-enhanced AuNPs that conjugated with PFOS-activated PPARα (Figure 3d).

Optical fiber sensors have shown advantages like low cost, real-time and remote monitoring, stability in natural environments, and simple surface modification. They have also been used to detect PFAS based on either Fabry–Perot interferometry (FPI)^55^ or SPR (Figure 3e).^56,57^ Polyvinylidene fluoride (PVDF),^55^ an ad hoc produced monospecific antibody,^56^ or molecularly imprinted polymers (MIPs)^57^ were coated on the optical fiber surface to increase the selectivity toward PFAS. The LODs of these optical fiber sensors range from 0.13 μg/L to 5 mg/L. Light scattering is another simple way to monitor PFAS concentration. Both resonance light scattering (RLS, elastic)^58^ and Raman scattering^59^ (inelastic) have been exploited in sensor design. In both cases, cationic dyes (e.g., Janus Green B,^58^ ethyl violet, or methyl blue^59^) were introduced in the system to complex with PFAS, enhancing the optical signal (Figure 3f). The LODs of these two sensors were 2.8 μg/L for the RLS sensor^58^ and 50 μg/L for the Raman scattering sensor.^59^

Recently, electrochemical sensors have also been developed for PFAS detection. Although there are not as many examples of these sensors compared to optical sensors, they are attracting increasing attention due to their ultrahigh sensitivity, simplicity, low cost, and fast response. Current electrochemical sensors for PFAS use voltammetry, impedance spectroscopy, or potentiometry as the measurement tool. In voltammetry-based sensors, the role of PFAS could be as blocking agents for electroactive species,^60,61^ transferable ions,^62^ or surfactants.^63^ Karimian et al. coated a gold electrode with a layer of MIP using PFOS as the template molecule.^60^ This MIP provides customized vacancies for PFOS binding. The electroactive probe, ferrocenecarboxylic acid (FcCOOH), could access the electrode surface through vacancies in the absence of PFOS molecules and undergo redox reaction, generating current signals. When PFOS are present, they would occupy the vacant sites in the MIP, thus blocking FcCOOH from reaching the electrode surface and reducing the current density in voltammograms. Based on this mechanism, this sensor was able to detect PFOS concentration with a LOD of 20 ng/L (0.04 nM). Utilizing a similar mechanism, Glasscott et al. designed a MIP-modified microelectrode to detect GenX and achieved ultrasensitive detection with a LOD of 82.5 pg/L (250 fM) (Figure 3g).^61^ Garada et al. used ion-transfer voltammetry to measure the lipophilicity and fluorophilicity of perfluoroalkanesulfonates and perfluoroalkanecarboxylates as well as to detect the concentration of PFOS ions. They employed poly(vinyl chloride) (PVC) membranes plasticized with 2-nitrophenyl octyl ether (oNPOE) as a selective membrane for PFAS ions.^62^ By correlating the peak current intensity in the ion-transfer voltammetry with PFOS ion concentration, they achieved a detection limit for PFOS at 25 ng/L (50 pM) after a 30 min preconcentration step.

Ranaweera et al. designed an innovative method for PFAS detection utilizing the surfactant nature of PFAS.^63^ The presence of PFAS as surfactants would stabilize gas nuclei produced by the hydrogen evolution reaction (HER) and thus lower the barrier for bubble formation, and this phenomenon could be transformed into changes in electrochemical signal and be correlated to PFAS concentration. By incorporating a 1000-fold preconcentration step using SPE, the LOD of this sensor for PFOS could achieve ∼40 ng/L. Recently, Cheng et al. demonstrated an impedance-based electrochemical sensor for PFOS (Figure 3h).^64^ They synthesized a mesoporous metal–organic framework (MOF) probe, which had high affinity toward PFOS, and then embedded it into a microfluidic channel. The capture of PFOS molecules in the MOF probe led to the increase in the impedance, the degree of which could be related to PFOS concentrations in sample solutions. Using the selective PFOS capture probe, the special electrode configuration, and the application of a microfluidic platform, this sensor achieved a LOD of 0.5 ng/L. Chen et al. reported a potentiometric detection method for PFOA and PFOS ions using ion-selective electrodes (ISEs) (Figure 3i).^65^ The response for this type of sensor is driven by ion exchange activity (in the case of PFOA and PFOS, it is driven by anion exchange). Fluorophilic anion exchangers were incorporated into a fluorous sensing membrane embedded in the electrodes to form a selective probe for PFOA and PFOS. These electrodes exhibited Nernstian response, with a decrease of 59.2 mV per 10-fold increase of the PFOA/PFOS ion concentration. This method has a LOD for PFOA at 70 ng/L and for PFOS at 430 ng/L with additional conditioning steps.

Photoelectrochemical sensors and electrochemiluminescence (ECL) sensors combine optical and electrochemical mechanisms together. Photoelectrochemical sensors are activated by light and produce electrochemical signals to reflect the analyte information, while ECL sensors are initiated by electrochemical reaction and eventually generate luminescence. For PFAS detection, most photoelectrochemical sensors were designed in a similar manner as the voltametric sensors that used PFAS as the blocking agent for electroactive species (Figure 3j).^65,67^ The difference here is that the photocurrent caused by the photoactive species excited by light is the major source of current, while the electroactive species acts as an amplifier for the total current. The presence of PFAS suppresses the current contributed by electroactive species by blocking their path to the electrode, thus decreasing the total current intensity. Li et al. made this kind of sensor portable and disposable by using screen-printed electrode technology (Figure 3j).^67^ Another photochemical sensor avoided introduction of electroactive species in the system by coating photoactive titanium oxide (TiO_2_) surfaces with a MIP and allowing PFAS to interact with the MIP to change the photocurrent.^68^ This sensor showed an increasing current intensity with increasing PFOS concentration, but the mechanism for this sensor is not yet fully understood. In an ECL sensor for PFAS, PFOA was designed to be a quencher for the ECL system by consuming one of the coreactants that is essential for the luminescence (Figure 3k).^69^ The LOD of the mixed sensors mentioned above are generally around 0.01 μg/L,^65,67,69^ except that the TiO_2_-based photochemical sensor has a lower sensitivity with a LOD of 86 μg/L.^68^

These emerging PFAS sensors not only show the diversity of possible ways to achieve simple detection of PFAS, but many of them also demonstrate on-site applicability by offering sufficient sensitivity, promising selectivity, and potential portability. Despite all this progress, PFAS sensors are still an immature and growing area, facing many challenges toward application. For example, the complexity of real water samples will hamper the sensitivity. In one fluorescence sensor, for example, the LOD of the device for PFOS increased from 10.7 μg/L (21.4 nM) to 60.4 μg/L (120.8 nM) when the sample matrices changed from buffer solution to real lake water.^30^ Therefore, preconcentration and pretreatment are typically required for PFAS sensors. Solid-phase extraction is the standard method for preconcentration used by the EPA,^26,27^ and it has also been frequently applied in various sensing devices.^42^ Recently, Cao et al. invented a rapid and convenient preconcentration method based on electrochemical aerosol formation. They achieved 1000-fold enrichment of 10 common PFAS in 10 min at the concentration range of pM to nM.^70^ While the pretreatment method is improving, how to integrate it with the sensing device and how to optimize the process for on-site use are also important considerations. Selectivity is another key issue to be addressed in order to achieve practical application. For PFAS sensors, selectivity should be compared not only with other organic or inorganic interferents in water but also within the PFAS family itself, and this requires a more subtle design for PFAS-capturing probes based on a deeper understanding of the PFAS–probe interactions. Meanwhile, considering the abundance of PFAS members and the requirement for efficient detection, a sensor that enables multiple-analyte detection at the same time is essential. This need also calls for better probe design as well as efficient data processing techniques. As the development of novel PFAS sensors progresses, standards for evaluating and comparing sensor performance will become increasingly important. Sensitivity, selectivity, and reliability are the most basic and crucial parameters of PFAS sensors. For the sensitivity or limit of detection of the sensor, guidelines provided by official agencies (e.g., USEPA) could be used as standard criteria, which are typically below 70 ng/L. A LOD at the μg/L level is acceptable for highly contaminated areas, but this will limit the versatility of PFAS sensors, as the PFAS concentrations in most water bodies are just a few ng/L or even lower.^18^ To expand the application range for PFAS sensors, efforts should be made toward a LOD at or below ng/L. Selectivity is crucial for sensors but hard to compare in the case of PFAS detection. This is not only because the interferents are numerous and researchers choose different targets for analysis but also because there is a lack of a standard quantitative parameter to evaluate selectivity. Identifying the most common competitive species (i.e., commonly coexisting with PFAS and having potential to interact with probe materials) in a specific water matrix by looking at existing databases or instrumental analysis of the real water samples would be a reasonable approach to specify experimental conditions that are close to the real circumstances. Only when those requirements are met can we fairly and meaningfully compare the selectivity among different sensors. Sensor reliability evaluates the accuracy of the measurement. This is often tested by comparing the concentrations calculated from the sensor signal with the spiked values or values obtained by some reliable instruments like LC-MS/MS. Using these methods, many studies demonstrated sensor reliability in terms of recovery rate, which was in the range of 90–110% for most PFAS sensors despite having different sensing mechanisms.^50,60,67^ It is worth mentioning that the recovery rate calculated based on spiked value may be affected by the accuracy of experimental operations like weighing and making solutions, as well as the laboratory materials used to store the sample solutions, as many studies indicated that PFAS losses could happen through adsorption by laboratory materials including glassware and various polymer composites.^71,72^ Besides the three figures of merit mentioned above, other parameters like response time, cost effectiveness, and portability are also important to evaluate sensor performance. However, there are a limited number of studies on PFAS sensors that mention or demonstrate those properties.^30,64,65,67^ Many sensors are advertised as “real-time”, but usually there are limited quantitative data provided regarding the specific response time. Even when fast response is demonstrated, there may be time required to prestabilize the sensor signal, which may take several hours.^64^ In the future, researchers are encouraged to provide more information about the response time and cost-effectiveness of PFAS sensors in a quantitative and comprehensive manner. To demonstrate portability, a full setup of the sensing system could be shown and evaluated.

## 3 PFAS Sorbents

Adsorption is an environmentally friendly and versatile method to remove water contaminants, including PFAS. For PFAS adsorption, granular activated carbon (GAC) and anion-exchange resins (AERs) are the two most mature materials. However, pristine GAC and AER lack sufficient selectivity for PFAS, and their performance is easily hindered by other coexisting contaminants in water. Several classes of novel sorbents are emerging. Those novel sorbents are designed by either modifying traditional commercially available sorbents like AC and AER, adding functionality to natural sorbents, or synthesizing new advanced materials.^36^ Generally, PFAS sorbents can be grouped into four classes according to the substrate materials: carbonaceous materials, anion-exchange resins, inorganic materials, and polymers (natural and synthetic).

**Figure 4:**
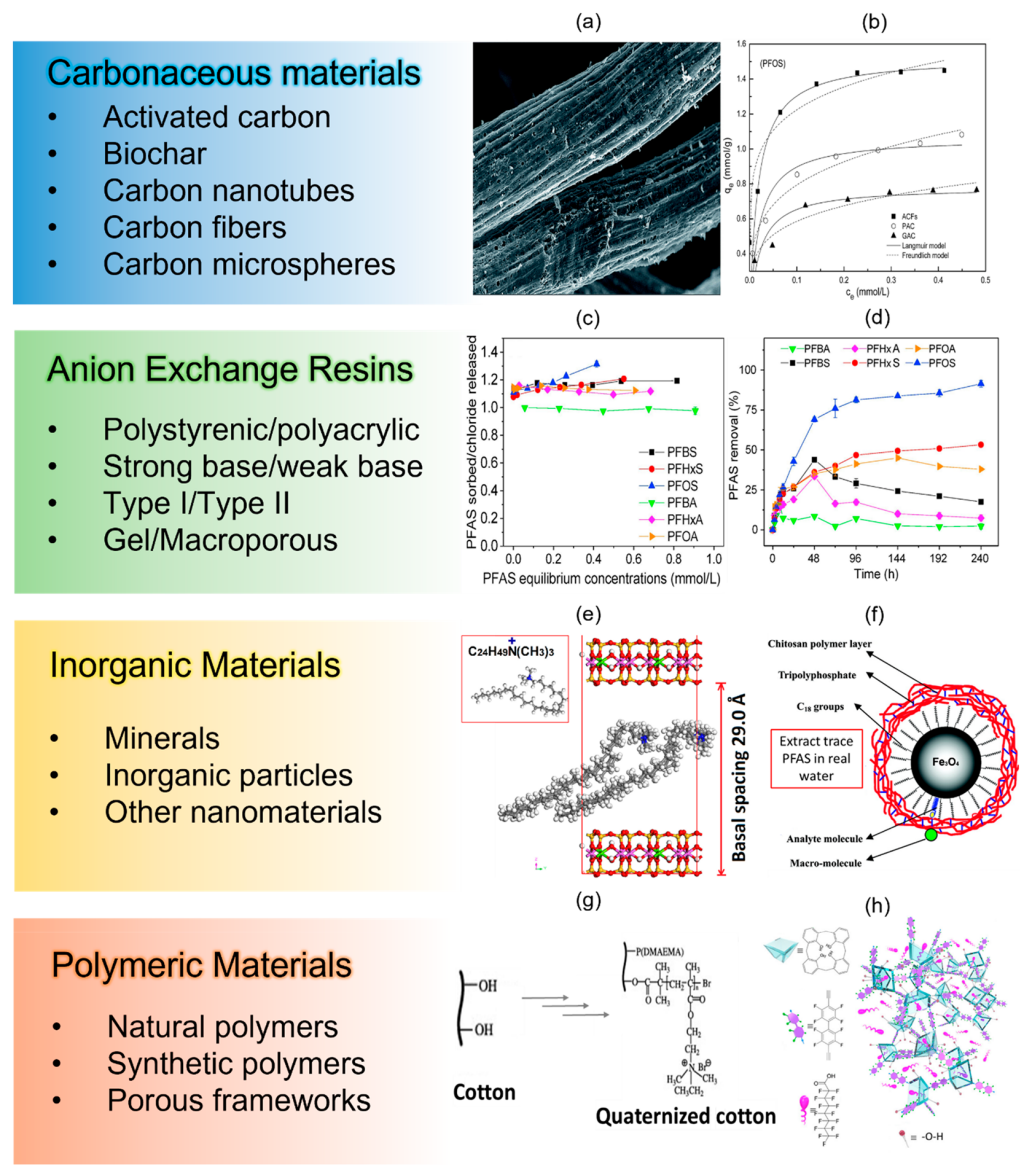
PFAS sorbent classification based on material classes. (a) SEM image of PACFs and (b) the sorption isotherm of PFOS and PFOA on the PACFs, PAC, and GAC. Reprinted with permission from ref (78). Copyright 2017 the Royal Society of Chemistry. (c) Ion exchange stoichiometry of PFAS on IRA910 (a commercially available AER) and (d) sorption kinetics of a mixed six PFAS molecules on IRA910. Reprinted with permission from ref (86). Copyright 2018 Elsevier. (e) A clay-based PFAS sorbent modified by intercalating [C_24_H_49_N^+^(CH_3_)_3_] into the interlayer spacing of the mineral sheets. Reprinted with permission from ref (91). Copyright 2020 Elsevier. (f) Chitosan-coated octadecyl-functionalized magnetite nanoparticles used for extraction of trace PFAS in real water samples with the anti-interference ability. Reprinted with permission from ref (100). Copyright 2010 American Chemical Society. (g) Quaternized cotton for efficient PFAS removal. Reprinted with permission from ref (105). Copyright 2012 Elsevier. (h) Synthetic fluorinated calix[4]arene polymers for PFOA removal. Reprinted with permission from ref (110). Copyright 2020 American Chemical Society.

Carbonaceous sorbents include GAC,^73^ powdered activated carbon (PAC),^74^ biochar,^75^ single-walled carbon nanotubes (SWNTs),^76^ multiwalled carbon nanotubes (MWNTs),^77^ carbon fiber,^78^ and carbon microspheres (CMSs).^79^ GAC and PAC, as the commercially available sorbents, are economic, scalable, and eco-friendly, but their application has been limited by the insufficient selectivity and slow adsorption kinetics. The adsorption capacity of these carbon-based sorbents varies from several mg/g (PFAS/sorbent) to several hundreds of mg/g.^36^ Under comparable conditions, PAC has a larger adsorption capacity than GAC;^78^ microporous AC has a higher adsorption capacity than SWNTs; and SWNTs have a higher adsorption capacity than MWNTs.^80^ This difference in adsorption capacity is largely dominated by the Brunauer–Emmett–Teller (BET) surface areas of the sorbents.^76^ The morphology of carbon materials also plays an important role in the adsorption performance. For example, Chen et al. fabricated polyacrylonitrile fiber (PANF)-derived activated carbon fibers (PACFs) and achieved an adsorption capacity for a PFOA of 302.3 mg/g and PFOS of 760.2 mg/g, which is 1.5 to 2.5 times higher than those of PAC and GAC (Figure 4a,b).^78^ In order to increase the selectivity and improve the adsorption performance of carbonaceous sorbents, a series of material engineering methods have been explored. Common sorbent modification strategies include introducing surface functional groups by oxidation,^81^ mixing with polymeric substrate,^82^ loading with metal oxide nanoparticles,^77^ and coating with MIPs.^79^

AER is an effective sorbent for PFAS with generally higher adsorption performance than activated carbon, usually having adsorption capacity over 1000 mg/g.^36^ Its adsorption capacity and rate mainly depend on the polymeric substrate, the functional group, and the porosity of the resin.^83^ According to these three properties, Boyer et al. classified AER into several subgroups.^84^ Based on the polymeric matrix, AER could be divided into polystyrene (PS) type and polyacrylic (PA) type, where PS type is more hydrophobic according to their chemical structures. Resins that have quaternary ammonium functional groups are called strong-base AER (SB-AER), while those having tertiary amine groups are called weak-base AER (WB-AER). The latter show pH-dependent charging behavior, whereas the former have a permanently charged group. According to the chemistry of the alkyl chain attached to the amine, SB-AER could be further categorized into Type I and Type II, where Type II is more hydrophilic due to the hydroxyl group at the end of the alkyl chain.^84^ The length of the alkyl chain is also tunable, changing the hydrophobicity and steric effects of the sorbents.^85^ Based on the porosity, AER could either have gel pore structure or macroporous structure, where gel-type AER has an additional size-exclusion effect compared to the macroporous type. The ion exchange stoichiometry is often larger than one (Figure 4c), which means for one ion exchange site there could be multiple PFAS molecules adsorbed, and this could not be achieved by solely ion-exchange activity as the charges are not balanced in this case. This indicates that, beyond electrostatic interaction, nonelectrostatic interaction, including hydrophobic interaction and van der Waals interaction, would synergistically impact the PFAS adsorption behavior. Because of the presence of nonelectrostatic interactions, predominantly hydrophobic interactions, the removal performance of AER exhibits a positive correlation with increasing PFAS length (Figure 4d)^86^ as longer chain length often leads to higher hydrophobicity for PFAS.^36,43^ These synergistic effects of different interactions have improved the adsorption performance for AER compared to other kinds of sorbents, especially for the adsorption of short-chain PFAS.^36^

Inorganic materials, including natural minerals and inorganic nanoparticles, have also been utilized for PFAS adsorption. Examples of natural minerals that have been tested as PFAS sorbents include alumina,^87^ silica,^87^ montmorillonite,^88^ kaolinite,^88^ hematite,^88^ calcined hydrotalcite,^89^ zeolite,^90^ sodium bentonite,^91^ and boehmite.^92^ The complexity of the mineral structures renders a combined adsorption mechanism with multiple interactions: electrostatic interaction, surface complexing, hydrogen bonding, steric confinement, and ligand exchange.^88,90,93^ The adsorption capacity of mineral sorbents, however, is limited to below 10 mg/g for most cases;^44^ this may be due to the hydrophilic nature of most minerals, making it hard to adsorb hydrophobic molecules.^94^ One strategy is to intercalate cationic surfactants into the interlayer spacing of the minerals, enhancing hydrophobic and electrostatic interactions (Figure 4e).^91,95^ Grafting amine functional groups^96^ or fluorinated alkyl chains^97^ onto mineral surfaces can also improve the adsorption performance. Nanoparticles like silica, iron oxides, titanium oxides, and alumina have a high surface area and many reactive sites and have also been examined for PFAS removal.^98^ Instead of acting as a PFAS sorbent alone, nanoparticles are more often mixed with other types of sorbents to improve the overall adsorption behavior.^99−101^ For example, Zhang et al. fabricated silane-functionalized, chitosan-coated magnetite nanoparticles (MNPs) to extract PFAS from real water samples (Figure 4f). The coatings not only improved the dispersibility of MNPs in water but also increased the anti-interference ability of the adsorbent against natural organic macromolecules in complex water samples by size exclusion and electrostatic repulsion.^100^ Other nanostructured inorganic materials such as modified titanate nanotubes^102^ and magnetic mesoporous carbon nitride^103^ also showed reasonable adsorption capacity for PFAS owing to their large surface area and PFAS-attracting functional groups.

Polymers are a class of powerful materials for water purification, not only due to their abundance in natural sources but also because of their superior flexibility in structural design and excellent tunability in chemical and physical properties. Polymeric materials have also been extensively studied for PFAS adsorption, ranging from modified natural polymers to various synthetic polymers. For naturally occurring polymers, cyclodextrins,^40^ chitosan,^104^ cotton (Figure 4g),^105^ cellulose,^39^ and rice husk^106^ have been modified and tested as PFAS sorbents with decent adsorption capacity and selectivity reported. Cross linking is one of the most common modification methods. By tuning the cross-linker chemistry^100^ or using a molecular imprinting technique,^97^ selective adsorption could be achieved. Surface modification by chemical reactions to introduce amine-containing functional groups is another useful approach to increase PFAS affinity (Figure 4g).^39,105,106^ For synthetic polymers, a variety of hydrogels have been synthesized with structures containing either fluorous segments or cationic segments to selectively capture PFAS in water.^38,108,109^ In recent years, porous polymeric sorbents containing cavity-like structures have emerged, such as fluorine-rich calixarene-based porous polymers (Figure 4h),^110^ MOFs,^111^ and amine-functionalized COFs.^112,113^ The porous structure could provide the sorbent with a large surface area and additional steric interaction for PFAS binding, which are favorable features for PFAS adsorption.

Overall, the performance of PFAS sorbents largely depends on sorbent properties as well as PFAS properties. Particle size, surface area, porosity, and surface chemistry are the most important factors that impact capacity and kinetics.^43^ PFAS with different chain lengths and headgroups have different adsorption behavior. Gagliano et al. compared the adsorption capacity of a wide range of sorbents for both short-chain PFAS and long-chain PFAS and found that capacity is always higher for long-chain species, indicating challenges for short-chain PFAS adsorption.^36^ Du et al. reported that for various kinds of sorbents including AC, resins, silicas, zeolites, sediments, and sludges PFAS with sulfonic headgroups were observed to have higher adsorption capacity than that of PFAS with carboxylic headgroups with the same carbon numbers.^43^ The higher capacities might result from the higher hydrophobicity of the corresponding type of PFAS.^36,43^ It is reported that long-chain PFAS like PFOA and PFOS could form hemimicelles or even micelles on sorbent surfaces,^74^ and this phenomenon would either enhance the adsorption by inducing PFAS aggregation^76^ or harm the adsorption by pore blockage.^74^ Additionally, characteristics of the sample solution including pH, background inorganic content like Na^+^, Ca^2+^, and Mg^2+^, and natural organic matter (NOM) like humic acid, fulvic acid, and nonfluorinated surfactants would also play important roles in PFAS adsorption. Their impacts are complex, being either beneficial or detrimental depending on details of the specific case. Several review papers have discussed these effects thoroughly,^36,43,114^ which will thus not be described here in detail.

Although the field of PFAS adsorption has been active, the existing sorbents all have their limitations, and there is still much room for development. For example, AC and AER have a high adsorption capacity, but their selectivity toward PFAS is limited. While synthetic or engineered materials can be tuned to possess high PFAS-specific affinity, their synthetic cost, reusability, and scalability need to be further evaluated. Adsorption capacity, kinetics, selectivity, and reusability are important figures of merits to evaluate sorbent performance. Most research on PFAS sorbents showed a high removal percentage for certain PFAS, but this does not guarantee the effectiveness of the sorbents as the applied dosage of the sorbents and concentrations of PFAS differ in various papers. Using adsorption capacity could reduce the bias caused by different experimental conditions and make the comparison among various sorbents meaningful. Similar to sensor research, the selectivity of sorbents is crucial but hard to compare as it is greatly impacted by the experimental conditions. Furthermore, few papers of PFAS sorbents demonstrated and discussed the selectivity by comparing the adsorption performance between PFAS and its interferents individually; instead, most of them examined the selectivity as the anti-interferent ability by showing an unaffected PFAS adsorption performance in the presence of interferents. This method is not unreasonable in terms of demonstrating the selectivity, but at the same time, one should pay attention to the dosage of sorbents, the species of selected interferents, and the concentrations of all analytes in sample solutions and make sure the unaffected performance is not caused by overloading of sorbents or using unrealistic sample solutions. Since the adsorption performance is largely related to the initial concentration of PFAS analyte, all sorbents should be tested under environmentally relevant PFAS concentrations (ng/L to μg/L). However, most existing research conducted adsorption experiments in batch mode under unrealistic initial concentrations (>mg/L),^36,115^ which cannot truly reflect the capability of those sorbents in real applications. Furthermore, the performance of more sorbents, especially emerging ones, needs to be tested in the column mode, which is a closer proxy to practical environments.^115^ Regeneration of used sorbents is another challenging issue. Generally, current chemical solution-based washing requires the use of toxic organic solvents, which is not environmentally benign. Harmless washing solutions like ammonium chloride (NH_4_Cl) and ammonium hydroxide (NH_4_OH) commonly used in the industry may be ineffective for sorbents having strong binding with PFAS.^116^ Thermal regeneration at a high temperature brings a high energy cost and may also decline the adsorption capacity as well as release harmful byproducts into the environment.^36^ Therefore, better solutions to maintain the life cycles of the PFAS sorbents need to be designed. The consideration and examination of sorbent capacity, kinetics, selectivity, and reusability in sorbent design are inseparable as those four properties are intercorrelated and affected simultaneously by certain factors. For example, one would expect strong and selective interactions to exist between sorbent materials and PFAS molecules, which may bring desirable selectivity and favorable adsorption kinetics. On the other hand, strong interactions may cause difficulties in releasing the adsorbed PFAS from used sorbents to achieve reusability. Cost-effectiveness is another important aspect to be considered for the practical application of PFAS sorbents. Activated carbons (ACs) are considered to be cost-effective materials, but regeneration of ACs by thermal methods often incurs additional cost. AERs have a relatively high capital cost, and thus it is more desirable to regenerate and reuse the spent resins.^117^ For most synthetic sorbents, the cost-effectiveness is yet to be evaluated in terms of material synthesis, used sorbent disposal or regeneration, and operational cost.

## 4 Selectivity toward PFAS

### 4.1 Selectivity of PFAS Sensors and Sorbents

The selectivity of sensors and sorbents is a key factor affecting their performance because of the complexity of water matrices and the relatively low concentration of PFAS analytes in water. For PFAS sorbents, interfering species like inorganic ions and NOM compete with PFAS to occupy the limited binding sites. If sorbents lack specificity for PFAS, the PFAS adsorption capacity will be limited.^118,119^ For PFAS sensors, the requirement for selectivity is even more strict. A lack of selectivity will lead to malfunction, thus reducing the accuracy and effectiveness of the sensor. Furthermore, PFAS sensors ideally should not only be able to isolate influences from interferents outside the PFAS family but also have the ability to distinguish between different members from the PFAS family. Although the number of published reports on PFAS sensors is small, most have included a selectivity test, as this is a basic requirement for sensors. In contrast, there are abundant papers on PFAS sorbents, but the number of papers that demonstrate selectivity is limited. Generally, selectivity experiments can be conducted either in individual mode or mixed mode. Individual mode means there is only one analyte or interferent in the sample solution, and this mode can help better understand single-solute behavior. Mixed mode refers to an approach where analytes and interferents are mixed together in one solution, and this is beneficial to understand the synergistic effects of analytes and interferents, which is closer to the real circumstances in application. Both modes are worthwhile as they provide complementary information. Sometimes, interferents have different effects when tested in different modes such as by showing little response in one mode and an obvious influence in another.^79,104^ A common challenge is that selectivity studies have used various experimental conditions like initial concentrations of the solutes, pH, and adsorption time. Just as these experimental conditions will affect sensing signal or adsorption performance, they may also affect the selectivity of the sensor or sorbent. To the best of our knowledge, no one has yet systematically tested the selectivity under different conditions to see how those parameters would affect the selectivity. Some studies apply unrealistic conditions by using an equal concentration or even higher concentration of the PFAS analyte than interferents which in fact have much higher concentrations than PFAS in most practical situations. Using real water samples is one solution to reduce the deviation from practical cases. By extracting solutes from those real water samples, the selectivity in individual modes could also be tested.^30^

Examples of research that demonstrate the selectivity for PFAS sensors or sorbents are summarized below (Table 1 and Table 2). For PFAS sensors, the majority of them used MIPs as the selective probes; a few applied cavity-containing probes like guanidinocalix[5]arene^30^ and COFs^50^ or adopted linear hydrophobic probes like cationic surfactant cetyltrimethylammonium bromide (CTAB)^122^ and fluorinated alkanethiols.^51^ The analyte of their selectivity studies was mostly PFOS, followed by PFOA, with one study focusing on GenX.^61^ The concentrations of those PFAS analytes in most studies were quite high. If we take USEPA’s advisory level as a comparison, the concentration of PFOS or PFOA should be below 70 ng/L, which is 0.14 nM for PFOS and 0.17 nM for PFOA. However, many studies use several nM (>1 nM) and even μM or hundreds of μg/L as the concentration, which exceed the guidelines and most practical circumstances. This also applies to sorbent studies. Interferents chosen for selectivity experiments are diverse, including salts like NaCl, KCl, and Na_2_SO_4_; metal ions like Fe^3+^, Ca^2+^, and Mg^2+^; and organic matter like humic acid, sodium dodecyl sulfate (SDS), and sodium dodecylbenzenesulfonate (SDBS). However, this diversity brings some concerns. First, most studies lack an explicit explanation for the interferents and the concentrations they applied in the selectivity studies. To address this, one direct strategy is to choose a specific real water sample, analyze the sample composition using reliable instruments, and find out the most important interferents for a given PFAS target. Moreover, the varying experimental conditions make it hard to compare the selectivity between devices. Overall, this diversity makes it difficult to judge sensor selectivity fairly and meaningfully. This situation also exists for selectivity studies of PFAS sorbents. Both individual modes and mixed modes are commonly applied in the selectivity tests for PFAS sensors and sorbents. Some studies, however, used different interferents and concentrations when testing the individual modes and mixed modes.^44,68,99,122^

**Table 1:** Examples of PFAS Sensors with Demonstrated Selectivity

		concentration					
mechanism	selective probe	analytes	interferents	mode	signal	selectivity	ref
voltammetry	MIP (poly(*o*-PD))	GenX: 1 nM	NaCl: 1 mM	mixed	normalized peak current	unaffected	(61)
			HA: 1 ppm				
			PFOS: 1 nM				
voltammetry	MIP (poly(*o*-PD))	PFOS: 2 nM	DBSA, PFOA, PFHxS, PFHxA, HFBA, PFBS: 2 nM, 20 nM	mixed	normalized current	unaffected except with HFPA and PFBS (with 10-fold higher concentration)	(60)
impedance	MIP (poly(*o*-PD))	PFOS: 0.7 nM	Humic acid: 1 ppm	mixed	*R*_ct_–*R*_ct_^o^ (kΩ)	unaffected	(120)
			NaCl: 0.7 nM				
fluorescence	guanidinocalix[5]arene	PFOS, PFOA: 0.8 μM	CTAB, octanesulfonic acid, octanoic acid, perfluorohexane, NaCl, Na_2_SO_4_, KCl, MgCl_2_: 0.8 μM	individual	(*I* – *I*_0_)/*I*_0_	selective to all	(30)
			wastewater: 5 μg/mL				
fluorescence	surfactant-sensitized COFs	PFOS: 18 nM	PFHxS, PFDA, PFNA, PFOA, PFHpA, PFHxA: 18 nM	individual	*F*_0_ – *F* (a.u.)	selective to all	(50)
fluorescence	MIP (chitosan-based)-CQDs	PFOS: 0.05 M	**individual**	individual and mixed	*K*_sv_, *F*/*F*_0_	*K*_sv_ of PFOS is higher than interferents, unaffected	(44)
			PFBS, OSA, PFSF, PFOA, SDBS, SDS, SDS′: 0.05 M				
			**mixed**				
			Na^+^, Fe^3+^ Mg^2+^, Ca^2+^, lactose, glucose, HAS: 0.05 M				
fluorescence	MIP, APTS	PFOS	PFOA, PFHxA, PFHxS, phenol, SDBS	individual	quenching constant *K*_sv_	*K*_sv_ of PFOS is higher than interferents	(121)
fluorescence	CTAB	PFOA, PFOS: 2 μM	**individual**	individual and mixed	*F* – *F*_0_, FL intensity (a.u.)	selective to all, unaffected	(122)
			PFPrA, PFBA, PFPeA, PFHeA, PFHpA, PFDeA, PFBS, SDBS, SDS, PFO and HFB: 2 μM				
			**mixed**				
			Mg^2+^, NH_4_^+^, Ba^2+^, Na^+^, NO_3_^–^, CH_3_COO^–^, Cl^–^: 100 μM				
			Mn^2+^, Ca^2+^: 50 μM				
			SDS, Ag^+^: 30 μM				
			Fe^3+^, Zn^2+^, Al^3+^, Tb^3+^, Cd^2+^: 10 μM				
			SDBS, Eu^3+^, La^3+^: 3 μM				
absorbance	Fe_3_O_4_ NPs	PFOS: 12.5 μM	PFOA, Fe^2+^, Mg^2+^, CO_3_^2–^, HCO_3_^–^, K^+^, Ca^2+^, NH_4_^+^, Na^+^, Br^–^, H_2_PO_4_^–^, HPO_4_^2–^, SDS, SDBS: 12.5 μM	individual	absorbance	selective except to PFOA, Fe^2+^, Mg^2+^, SDS, SDBS	(53)
absorbance	fluorinated alkanethiol	PFOS: 500 μg/L	octanoic acid, decanoic acid, dodecanoic acid, 1,2-dodecandiol, 1-dodecylamine, 1-hexadecylamine, SDS, SHDS, SODS, SDBS, CTAB, PFOA: 500 μg/L	individual and mixed	absorbance intensity (a.u.)	selective to all except PFOA, unaffected	(51)
			NaCl, MgCl_2_, CaCl_2_: 500 mM				
photoelectrochemistry	MIP (cross-linked polyacrylamide)	PFOSF: 50 ng/mL	2,4-D, PCP, MP, OA, PFPeA, PFHPA, PFNA, PFOS, PBSF: 50 ng/mL	individual and mixed	relative photocurrents	selective to all, unaffected	(67)
photoelectrochemistry	MIP (cross-linked polyacrylamide)	PFOA: 100 ng/mL	OA, DA, 2,4-D, PCP, OTAB, PFPA, PFHA, PFHpA, PFNA, PFDA, PFOS: 100 ng/mL	individual and mixed	relative photocurrents	selective to all, unaffected	(65)
photoelectrochemistry	MIP (cross-linked polyacrylamide)	PFOS: 5 μM	**individual**	individual and mixed	relative photocurrents	selective to all, unaffected	(68)
			2,4-D, 9-AnCOOH, PCP, PFHA, PFOA: 5 μM				
			**mixed**				
			2,4-D, 9-AnCOOH, PCP: 100 μM				
			PFHA, PFOA: 10 μM				
electrochemiluminescence	MIP (polypyrrole)	PFOA: 100 ng/mL	PCP, 2,4-D, MP, PFPA, PFVA, PFHA, PFHpA, PFNA, PFDA, PFOS: 100 ng/mL	mixed	ECL intensity (a.u.)	unaffected	(69)

**Table 2:** Examples of PFAS Sorbents with Demonstrated Selectivity

		Concentration					
material class	selective probe	analytes	interferents	mode	parameter	selectivity	ref
mineral	all-silica zeolite beta	PFOA, PFOS: 100 μM	CA, SDS, BA, AA, phenol:100 μM	mixed	*q* (mg PFAS/g)	unaffected	(90)
mineral	PFQA	PFOA, PFOS: 5 μmol/L	SDBS, pyridine, PHE, phenol: 5 μmol/L	individual and mixed	adsorbed amount	selective to all, unaffected	(97)
inorganic nanoparticle	fluorous/amine groups	PFHxS PFOS	**individual**	individual and mixed	equilibrium sorption amount, total removal efficiency	PFCs showed good selectivity to interferents, unaffected	(99)
		PFHpA PFOA	6:2 FTOH, *n*-OA, 2-FPAA, 3,4-DHPAA, FTAP, ERY: 0.18 mg/mL				
		PFNA PFDA	**mixed**				
		PFUnDA PFDoDA PFTA: 0.18 mg/mL(individual), 5 ng/L (mixed)	HA: 5, 10, 20, 50 mg/L				
polymer	fluorous-core nanoparticles	PFOA: 1 μg/L to 10 mg/L	inorganic salts: 100 mg/L	mixed	adsorption capacity, removal efficiency	adsorption capacity is moderately decreased; removal efficiency is unaffected	(123)
			decanoic acid: 20 mg/L				
polymer	quarternary amine group	PFBA, PFPEA, PFHXA, PFHPA, PFOA, PFBS, PFPES, PFHXS, PFHPS, PFOS, GenX: 1 μg/L or 5 μg/L	Cl^–^, SO_4_^2–^: <1 mg/L to 150 mg/L	mixed	removal efficiency	PFOA, PFOS, PFHPS, and PFHXS are unaffected, while others are affected to a varying degree	(109)
			NO_3_^–^: <1 mg/L to 50 mg/L				
			DOC: 2.5 mg/L to 5 mg/L				
polymer	PEI-*f*-CMC	PFBA, PFPeA, PFHxA, PFHpA, PFOA, PFNA, PFDA, PFDoDA, PFTrDA, PFBS, PFPeS, PFHxS, PFHpS, PFOS, PFNS, PFDS, ADONA F-53B, 4:2 FTS, 6:2 FTS, 8:2 FTS, *N*-MeFOSAA, *N*-EtFOSAA: 1 μg/L	DOC = 2 mg/L	mixed	removal efficiency	most are slightly affected or unaffected except PFBA, PFPeA, PFHxA, and PFPeS, 4:2 FTS	(39)
polymer	DFB-CDP	PFOA: 1 μg/L	humic acid: 20 mg/L	mixed	removal ratio	unaffected when adsorption time >5 h	(40)
polymer	fluorous/amine/ammonium segments	PFOA, PFOANH_2_, PFHxA: 10 ppm	OA, HxA, NaBr: 10 ppm	mixed	removal efficiency	unaffected	(108)
polymer	MIP (chitosan-based)	PFOS:100 μM	PFOA, 2,4-D, PCP, SDBS, phenol: 100 μM	individual and mixed	removal rate	selective to all, moderately decreased by phenol and PCP	(104)
carbon	MIP with fluorous groups and amine groups	PFOS: 50 μM	PFOA, PFHSB, PFKSB, F53–B, BPA, DBP, NP: 50 μM	individual and mixed	removal rate	selective to PFOA and DBP	(79)

In contrast to PFAS sensors, selective probes incorporated into PFAS sorbents are more varied. Minerals, MIPs, fluorinated probes, cationic probes, or the combination of multiple probes have all been used to increase the PFAS specificity. A wider range of PFAS species have been tested in PFAS sorbent studies compared to predominately PFOS and PFOA in sensor studies. To distinguish different PFAS species within the PFAS family is a challenge due to the abundance of PFAS molecules and their structural similarities. A limited number of papers demonstrated the selectivity of PFAS analyte against PFAS interferents.^44,50,60,61,65,67−69,79,104,121,122^ Among them, most used MIPs as the selective probes to capture the specific PFAS target against other PFAS species. The tailored cavities and sometimes the chemistries of MIPs provide excellent accommodation ability for one certain PFAS, thus making it possible to achieve selectivity within the PFAS family. However, this selectivity is still not perfect and could be limited by smaller species with similar shape and chemistry to the PFAS analyte.^60^ Beyond MIPs, one study utilized surfactant-sensitized COFs as the probe and demonstrated selectivity toward PFOS against PFHxS, PFHxA, PFHpA, PFOA, PFNA, and PFDA.^50^ These researchers ascribed the possible reasons for this selectivity to the importance of the sulfonate group and the hydrophobic chain length. Another study used cationic surfactant CTAB as the probe.^122^ Despite the promising selectivity results shown, further study is needed to explain how the selectivity against so many structurally similar PFAS is achieved with a common cationic surfactant. Overall, achieving selectivity between different PFAS is still a grand challenge. To move forward, PFAS-related interactions need to be studied more precisely and comprehensively. Whenever a new promising material appears to exhibit selectivity, supporting theories could be proposed to explain the selective mechanisms based on either the experimental studies of PFAS interactions or computational simulations.

### 4.2 PFAS Interactions

To better understand the origin of PFAS selectivity, the interaction mechanisms between PFAS and selective probes, as well as the tools to characterize those interactions, will be discussed below.

#### 4.2.1 Characterization Tools

Recently, Liu et al. summarized the methods for characterizing PFAS–protein binding, which is important to understand cellular toxicities, biotransformation pathways, and the fate of PFAS inside the body.^124^ Those characterization tools include separative methods like size exclusion chromatography, spectroscopic methods like fluorescence, nuclear magnetic resonance (NMR), ultraviolet–visible (UV–vis) spectroscopy, and infrared (IR) spectroscopy, surfactant-based methods like using ion-selective electrodes, calorimetric methods like isothermal titration calorimetry (ITC) and differential scanning calorimetry (DSC), mass spectrometry (MS)-based methods like electrospray ionization (ESI) MS, and other methods such as surface plasmon resonance and molecular docking. Utilizing these methods, one can measure the binding affinity, binding kinetics and thermodynamics, binding stoichiometry, and binding site qualitatively or quantitatively. Although these methods were summarized for probing binding between PFAS and proteins, some could be or have already been used to measure the binding between PFAS and functional probes on sensors and sorbents.

Spectroscopic and computational methods are the two most extensively used tools to study analyte–material interactions in PFAS sensors and sorbents. For spectroscopy-based methods, NMR is one of the most powerful tools to measure the subtle changes in the environment around the analyte when an interaction happens. The multiple types and modes of NMR including standard NMR,^108^ variable-temperature (VT) NMR,^125^ diffusion-ordered spectroscopy (DOSY) NMR,^125^ nuclear Overhauser effect (NOE) difference spectroscopy,^108^ gradient heteronuclear multiple bond coherence (gHMBC) NMR,^126^ and heteronuclear Overhauser effect spectroscopy (HOESY) NMR^126^ enable us to extract multiple pieces of information about the interactions, such as binding geometry, binding ratio, and binding thermodynamics. Another category of popular characterization is X-ray-based scattering and spectroscopic methods. X-ray diffraction (XRD) is often used for characterizing crystalline mineral sorbents.^125,127^ The change in crystal structure upon adding PFAS could be an indicator of PFAS interaction. X-ray photoelectron spectroscopy (XPS)^64^ and X-ray absorption near-edge structure (XANES) spectroscopy^127^ have been used to detect the changes in elemental oxidation state induced by interactions with PFAS. Fourier-transform infrared spectroscopy (FTIR) also serves as a facile way to qualitatively probe the interactions. Garada et al. performed fragmental analysis based on potentiometry to quantitively measure the lipophilicity and fluorophilicity of PFAS.^62^ Complementary to experimental tools, computational methods and theoretical analyses are often applied to gain a deeper understanding of the interaction mechanisms.^30,90,91,97,109−111,125,127^ However, current computational studies typically explore simplified systems, and further improvement of these methods and simulated systems is essential to address the complexity of practical sensing and adsorption environments.

#### 4.2.2 Interaction Mechanisms

Current discussions on the interacting mechanisms for PFAS with their environment are mostly based on observations in adsorption experiments. Several reviews have analyzed and summarized such observations and concluded that the major adsorption mechanisms of PFAS include electrostatic interactions, hydrophobic interactions, ligand and ion exchange, and hydrogen bonding.^44,43^ Since most PFAS that are commonly found in the environment have a low acid association constant (p*K*_a_) value,^36^ they tend to exhibit anionic species at environmentally relevant pH values. It is worth noting that the p*K*_a_ values for many PFAS molecules are still under debate, and different databases sometimes give different p*K*_a_ values for the same PFAS. For example, according to one study, the p*K*_a_ for PFOS is 0.14,^128^ while another study reported a value of −3.27.^129^ The Hazardous Substances Data Bank (HSDB) (https://toxnet.nlm.nih.gov) gave a range of p*K*_a_ < 1.0 for PFOS.^36^ Despite the variations, the range of the p*K*_a_ values for those common PFAS is still low enough for PFAS to yield anionic species in natural water. If there are any positively charged groups on the material surface, PFAS could interact with them through electrostatic interactions. This kind of interaction could be affected by the pH and coexisting ions.^39,130^ The hydrophobic interaction is the affinity of nonpolar hydrophobes to originate from the entropic tendency of aggregation in aqueous solution and repulsion of water molecules.^131^ These interactions originate primarily between the fluorinated tail of PFAS and the hydrophobic moieties of other materials. Hydrophobic attraction was found to be enhanced with increasing C–F (fluorinated carbon) chain length.^43^ Strong hydrophobic interactions could lead to the formation of hemimicelles in the range of 0.01–0.001 times the critical micelle concentration (CMC) and even full micelles where the PFAS are enriched to a localized space.^74^ Similar to hydrophobic interactions, fluorophilic interactions are also proposed for PFAS binding as many studies showed that fluorinated probes had better adsorption performance than nonfluorinated ones.^97,132^ One study employed fragmental analysis and found out that the fluorophilicity of perfluoroalkanesulfonates is even higher than their lipophilicity.^62^

Ligand and ion exchange mainly occur when PFAS interact with AER or metal oxides,^43^ while hydrogen bonding could be formed between the polar headgroup of PFAS and the hydrogen atoms bonded to either nitrogen or oxygen in the functional groups on material surfaces. However, competitive hydrogen bonding with material surfaces will happen between water molecules and PFAS molecules, leading to insignificant contribution of hydrogen bonding PFAS sorption in some cases.^133^ Overall, these two kinds of interactions are less common than electrostatic interaction, hydrophobic interactions, and fluorophilic interactions and only happen in certain circumstances. The host–guest interaction is a composite interaction that involves two molecules or ions forming complexes through unique structural relationships and noncovalent binding. One special attribute of this interaction lies in the cavitary structure of host molecules. The size, shape, and chemistry of these cavities are often designed and tuned for a specific target.^134,139^

### 4.3 Selective Probes

Based on the understanding of PFAS–material interactions, a variety of selective probes have been designed and incorporated into PFAS sensors and sorbents. In the following, the application of those selective probes in PFAS sensors and sorbents will be introduced according to the functional groups they present. Fluorinated probes and cationic probes are the most extensively used structures and will be discussed both separately and synergistically. Cavitary probes, which can provide host–guest interactions and spatial confinement, will be discussed afterward. We hope these examples and discussion could provide insights into probe design toward future sensors and sorbents with enhanced selectivity.

#### 4.3.1 Fluorinated Probes

**Figure 5:**
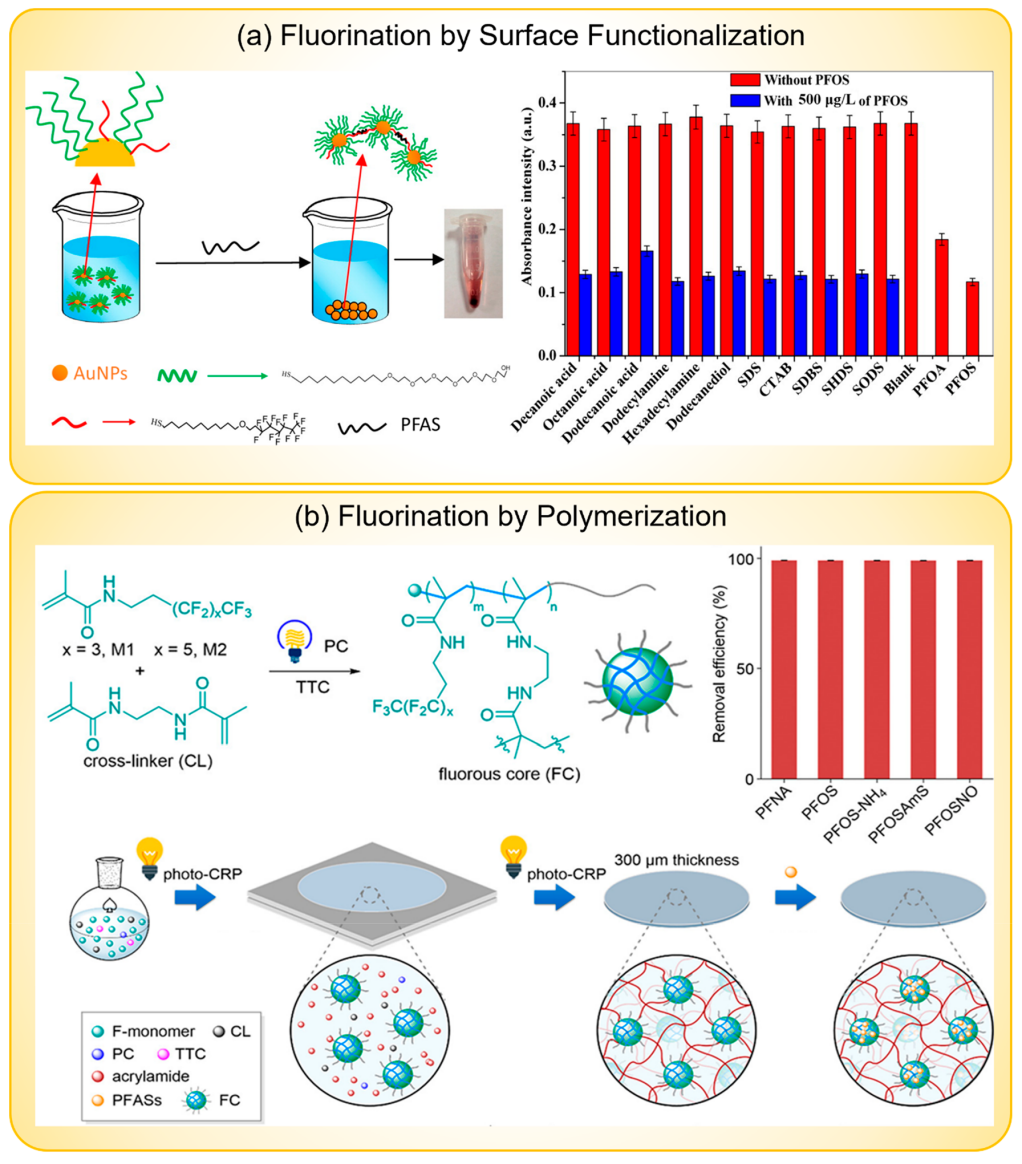
Examples of fluorinated probes. (a) A colorimetric sensor using F-thiol as the selective probe and exhibiting high selectivity toward several nonfluorinated organic interferents. Reprinted with permission from ref (51). Copyright 2014 American Chemical Society. (b) A fluorous-core nanoparticle-embedded hydrogel for PFAS removal with a high removal efficiency for neutral, anionic, cationic, and zwitterionic species. Reprinted with permission from ref (123). Copyright 2020 American Chemical Society.

Fluorinated probes utilize the selective nature of fluorophilic interaction to achieve selective binding with PFAS. One simple strategy to incorporate this kind of probe is by surface functionalization. Niu et al. developed a colorimetric sensor using a partially fluorinated alkanethiol (F-thiol) as the selective probe (Figure 5a).^51^ They fabricated the sensor (Au@PEG-F NPs) by modifying AuNP surfaces with poly(ethylene glycol)-terminated alkanethiols (PEG-thiols) and F-thiols. The role of PEG-thiol is to increase the dispersity of AuNPs in water. The minimum PEG-thiols to F-thiols ratio on AuNP surfaces they could achieve was 8.7, which is limited by the solubility of Au@PEG-F NPs. Upon interacting with PFAS, Au@PEG-F NPs would aggregate and precipitate from the solution, which led to a decrease in the absorbance of the sample solution, and this absorbance decreased linearly with increasing PFAS concentration. They used this method to detect 10 PFAS molecules of different chain lengths and headgroups and found that the sensor sensitivity increased generally with the elongation of CF_2_ and that sensitivity to perfluorinated sulfonates (PFSAs) is higher than perfluorinated carboxylic acids (PFCAs) with the same fluorinated chain length, which is consistent with the observation for other sorbents.^39,86,97,125,138^ The LOD for long-chain PFAS (perfluoroalkyl chain CF_2_ ≥ 7) was as low as 10 μg/L. They also demonstrated the selectivity of the sensor by measuring the response of 11 nonfluorinated organic interferents individually and simultaneously with PFOS at 500 μg/L and confirmed this sensor is highly selective to PFOS compared to those interferents. Additionally, they found that high concentrations (500 mM) of inorganic salts (NaCl, MgCl_2_, and CaCl_2_) have no influence on the PFOS recognition process. Similarly, Xiang et al. attached PFOA-like fluorinated probes and PEG-based probes to phenolic resin (PR) microsphere surfaces to achieve selective removal of PFAS.^135^ The modified PR microspheres were able to capture >90% of PFAS and showed selectivity against SDBS and SDS, which are two common interfering surfactants to PFAS. Interestingly, they utilized the thermally sensitive property of the PEG segment to achieve facile separation of the used sorbents from the sample solution.

Synthesizing polymers using fluorinated monomers is another feasible way to incorporate fluorous probes. Quan et al. designed and synthesized a fluorous-core nanoparticle-embedded hydrogel to capture different kinds of PFAS in water (Figure 5b).^123^ They first synthesized fluorous-core nanoparticles by photocontrolled living radical polymerizations (photo-CRPs) of fluorinated monomer (M) and cross-linker (CL) from a methoxy poly(ethylene glycol) (PEG)-substituted trithiocarbonate macroinitiator (TTC) using a photoredox catalyst (PC). Then, they investigated PFAS adsorption on the fluorous nanoparticles FC3 ([TTC]/[CL]/[M2] = 1:10:10) using ^19^F NMR. The results showed that FC3 exhibited strong interactions toward different types of PFAS including neutral, anionic, cationic, and zwitterionic species even under acidic/basic conditions or in the presence of inorganic salts and decanoic acid. They further embedded FC3 into hydrogels to form FCH sorbents. The adsorption performance of FCH was tested with various PFAS (i.e., PFOS, PFNA, PFOS-NH_4_, PFOSAmS, and PFOSNO) at an environmentally relevant concentration (1 μg/L). FCH exhibited a high removal efficiency around 99% and successfully reduced PFAS to less than 11 ng/L, lower than the USEPA’s advisory level.

#### 4.3.2 Cationic Probes

Most cationic probes used in PFAS sensors and sorbents are amine based. There is, however, at least one example using a phosphonium-based cationic surfactant as the anion exchanger in ion-selective electrodes for PFAS detection.^65^ Commercially available AERs are good examples of cationic materials based on ammonium groups.^84^ Ateia et al. summarized current research on amine-functionalized sorbents for PFAS removal,^115^ indicating a trend of incorporating amine-containing groups into PFAS sorbents to induce favorable electrostatic attraction as well as other accompanying interactions to realize effective PFAS removal. A study on the effect of functional groups on modified hexagonal mesoporous silica (HMS) for PFAS adsorption showed that the 3-aminopropyltriethoxy-modified HMS possessed both faster adsorption kinetics and higher adsorption capacity compared to pristine HMS, validating the positive effects of amino groups on PFAS adsorption.^96^ Amine groups exist in different forms when being incorporated into PFAS sorbents, including primary amino, secondary amino, tertiary amino, quaternary ammonium, aminopropyl, dimethylethanolamine, polyethylenimine, and polyamine/fatty amine.^115^ Those functional groups could be designed into materials by either surface modification or polymerization.

**Figure 6:**
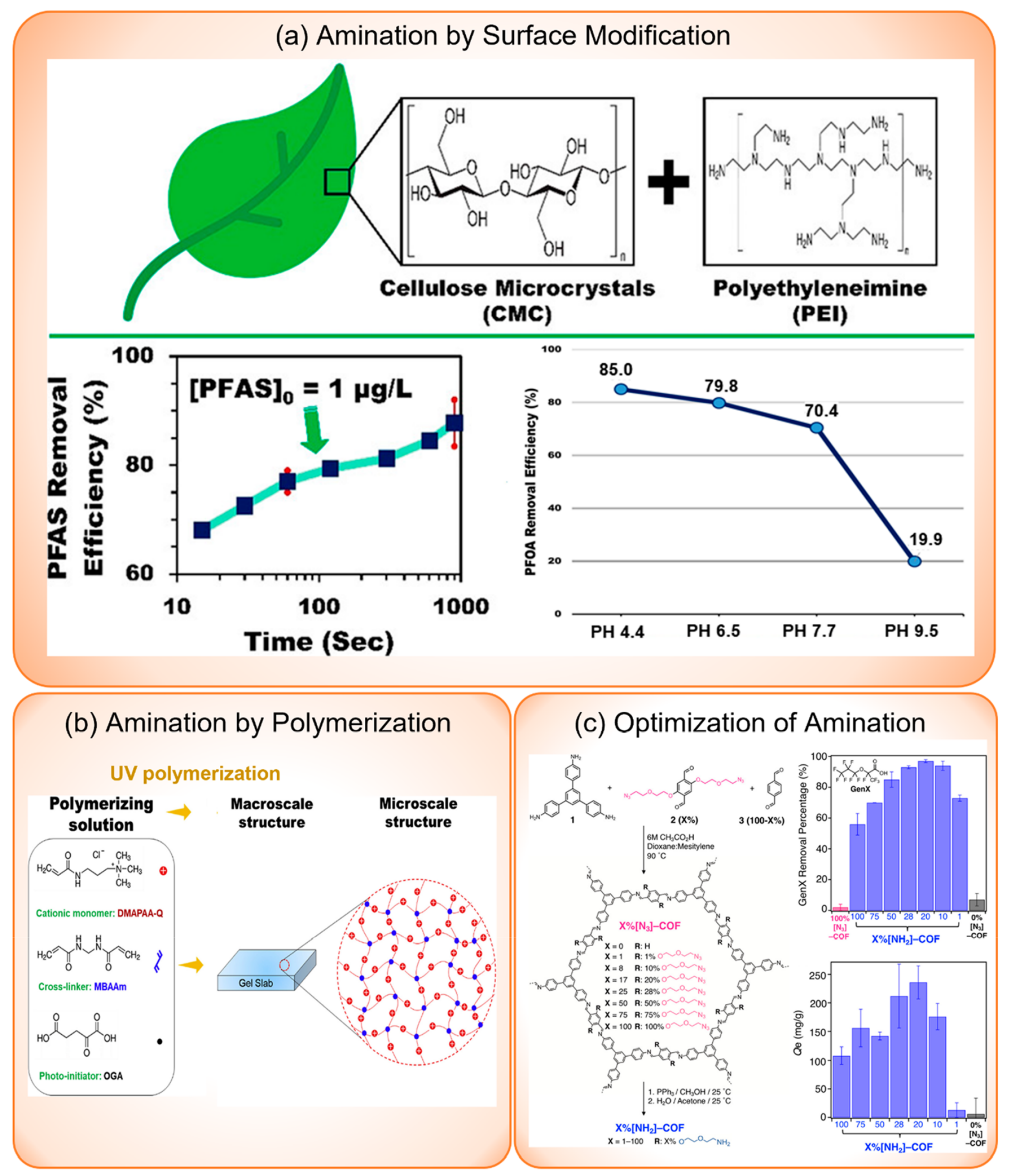
Examples of cationic probes. (a) A PEI-*f*-CMC sorbent showing fast adsorption kinetics and pH dependence. Reprinted with permission from ref (39). Copyright 2018 American Chemical Society. (b) A cationic polymer hydrogel containing quaternary ammonium for PFAS removal. Reprinted with permission from ref (109). Copyright 2019 Elsevier. (c) A porous COF-based sorbent bearing various amine loadings for GenX removal. Reprinted with permission from ref (112). Copyright 2018 American Chemical Society.

**Figure 7:**
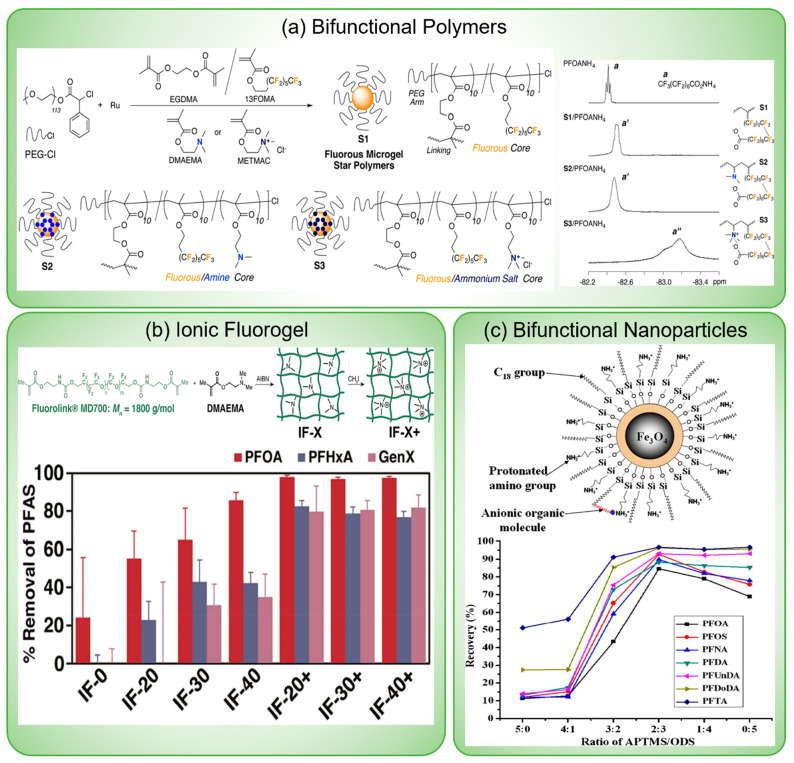
Examples of synergistic effects brought by the combination of fluorinated/hydrophobic probes and cationic probes. (a) Fluorous microgel star polymers with PEG arms synthesized by copolymerization of fluorous monomers with either amine-containing or ammonium-containing monomers, showing high affinity toward PFAS via synergistic interactions. Reprinted with permission from ref (108). Copyright 2014 American Chemical Society. (b) Ionic fluorogels with different contents of fluorinated backbones and amine functional groups for PFAS removal. *X* represents wt % of amine monomers. Reprinted with permission from ref (38). Copyright 2020 American Chemical Society. Further permission related to the material excerpted should be directed to the ACS. (c) Fe_3_O_4_/SiO_2_ magnetic nanoparticles functionalized with ODS and APTMS, achieving optimized PFAS adsorption at APTMS/ODS = 2:3. Adapted with permission from ref (137). Copyright 2011 Elsevier.

Ateia et al. functionalized cellulose microcrystals (CMCs) with poly(ethylenimine) (PEI) through TEMPO oxidation followed by ion exchange and amidation (Figure 6a).^39^ PEI is a polymer with many primary, secondary, and tertiary amino groups. The PEI-functionalized CMC (PEI-*f*-CMC) sorbent had a point of zero charge (pH_PZC_) at 10.9 ± 0.2, which means the material surface would be positively charged in solution pH values lower than 10.9. This sorbent exhibited fast adsorption kinetics, reaching equilibrium in ∼15 min with 70–80% removal within the first 100 s. The removal efficiency of PEI-*f*-CMC was examined against 22 PFAS molecules in deionized (DI) water and in lake water. The results showed that the removal efficiency increased with increasing chain length, and for those long-chain PFAS (C ≥ 8), the removal efficiency is close to or higher than 90%. The removal efficiency of most of the tested PFAS was not significantly changed even in the presence of high loading of NOM except for short-chain species like PFBA and PFPeA. It is noteworthy that the removal performance of PEI-*f*-CMC is affected by the pH value. When pH increased from 4.4 to 9.5, the removal efficiency for PFOA decreased from 85.0% to 19.9%. The authors explained that this decrease was attributed to the reduction in the number of positive sites on PEI-*f*-CMC when the pH value was elevated. In contrast, a PFAS sorbent based on quaternized cotton developed by Deng et al. through surface grafting quaternary ammonium-containing polymers P(DMAEMA) showed little dependence on the pH of solution (Figure 4g);^105^ this is because quaternary ammonium is a permanent cation that is not significantly affected by pH. Ionic fluorogels that incorporated quaternary ammonium (IF-*X*+) at various concentrations (*X* represents wt % of amine monomers) also showed a higher affinity toward PFOA, PFHxA, and GenX than those that incorporated tertiary amine (IF-*X*) at the same amine loading when pH = 6.4, indicating the importance of permanent charge in PFAS adsorption (Figure 7b).^38^

Similar to fluorinated polymers, cationic polymers can also be synthesized by using cationic monomers. Ateia et al. prepared a cationic polymer hydrogel (poly DMAPAA-Q hydrogel) by photopolymerization of a quaternary ammonium-containing monomer DMAPAA-Q with cross-linkers and photoinitiators (Figure 6b).^109^ They tested the adsorption performance of this cationic polymer toward 16 PFAS in distilled deionized water (DDI), lake water, the influent to a wastewater treatment plant (WTP), and treated wastewater and found that the removal efficiency in DDI of almost all tested PFAS could be maintained in lake water and the influent of a WTP, even when the concentration of dissolved organic carbon (DOC) in such water samples was 2.5 ± 0.3 × 10^6^ times higher than PFAS concentrations. However, the removal efficiency decreased by 10–20% in treated wastewater, which possessed lower specific ultraviolet absorbance and higher background anions than other samples. They also evaluated the influence of coexisting anions and DOC individually. The results showed that removal of long-chain PFAS was marginally influenced by increasing background anion concentrations (i.e., Cl^–^, NO_3_^–^, SO_4_^2–^), whereas the adsorption of short-chain PFAS significantly decreased with increasing anion concentrations. The removal efficiency for all tested PFAS dropped by 10–20% when the concentration of DOC increased to 5 mg/L (PFAS concentration was 1 μg/L).

Ji et al. fabricated amine-bearing porous polymers based on COFs (Figure 6c).^112^ The amine functionalities (NH_2_) were produced by the reduction of azides (N_3_) in one type of monomers. They prepared a series of *X*%[NH_2_]-COFs with different amine loadings, where *X*% represents the concentration of the amine group in COF sorbents, ranging from 0 to 100. The adsorption results showed that the removal percentage and adsorption capacity toward GenX reached a maximum value with 20% amine loading. This peak was explained by the interplay between the GenX affinity brought by amine functional groups and the steric hindrance induced by overloading amine. Even with relatively lower adsorption performance with 100% amine loading, the [NH_2_]-COFs still had a removal percentage close to 60%, much higher than that of azide-functionalized COFs (*X*%[N_3_]-COF), which exhibited limited removal (<10%) for GenX.

#### 4.3.3 Synergistic Effects of Fluorinated and Cationic Probes

In many cases, fluorinated probes and cationic probes have been combined together in one adsorption system to enhance PFAS affinity through synergistic effects and an interplay between fluorophilic and electrostatic interactions. Koda et al. synthesized a series of fluorous microgel star polymers with PEG arms by copolymerization of fluorous monomers with either amine-containing or ammonium-containing monomers (Figure 7a).^108^ They first studied PFAS recognition processes on those fluorous microgels using ^19^F NMR. The results revealed broader shape and stronger shifts of the fluorine peak of bifunctional microgels compared to only fluorous microgels, indicating cooperative recognition by fluorine–fluorine interaction and that electrostatic interaction would enhance the efficiency and selectivity of molecular recognition of PFAS. By comparing the recognition behavior of the fluorous star polymer developed in that work with fluorous star polymers with PMMA arms (more hydrophobic than PEG) and a conventional macroscopic gel comprising short PEG chains and perfluorooctyl pendants, they found the key to efficient PFOA recognition is creating a highly fluorinated and totally soluble nanoscopic gel network in water by condensing fluorinated groups into the microgel cores of the star polymer. In the following adsorption tests, they demonstrated that the star polymers with only fluorinated functionalities were sufficient for PFOA and PFOANH_2_ (removal efficiency close to 100%) even in the presence of octanoic acid (∼10 ppm) and NaBr (∼10 ppm). However, the removal efficiency for short-chain species PFHxA increased by ∼74% through exploiting the dual-recognition mechanism. Other bifunctional hydrogel-based PFAS sorbents developed by Huang et al.^136^ and Kumarasamy et al. (Figure 7b)^38^ also indicated an enhancement in adsorption affinity toward PFAS when fluorous probes and cationic probes are combined compared to that of single-type probes. The interplay between hydrophobic and ionic interactions was studied by Zhang et al.^137^ by modifying the surface of Fe_3_O_4_/SiO_2_ nanoparticles with mixed groups of octadecyltriethoxysilane (ODS) and aminopropyltrimethoxysilane (APTMS), which are a long alkyl chain and a short alkyl chain terminated with the amine group, respectively (Figure 7c). By varying the ratio between ODS and APTMS, they observed optimized adsorption at APTMS/ODS = 2:3 for PFOA, PFOS, and PFNA. Further increasing the APTMS/ODS ratio was detrimental to the adsorption. However, for PFAS with longer chains, the adsorption would not decrease with increasing APTEMS/ODS ratio but rather was stable after it reached a maximum performance at 2:3. These results indicated that hydrophobic interactions dominated for adsorption of long-chain PFAS, whereas ionic interactions were more important for PFAS with shorter chains.

Li et al. innovatively incorporated fluorous probes and cationic probes by grafting oxidized fluorinated graphene (OFG) onto amino-terminated alkyl-chain-modified silica (OFG@silica), rendering synergistic effects among hydrophobic, fluorophilic, and electrostatic interactions.^138^ The adsorption behavior and mechanism of OFG@silica toward PFAS were explored by packing it into an SPE cartridge and flowing a sample solution through the cartridge, followed by flowing a washing solvent. The results showed that OFG@silica had a relatively high adsorption amount and recovery toward PFBA, PFOA, and PFTeDA compared to interfering species like 1,2-DAB, aniline, phenol, 2,4-DCP, toluene, and naphthalene. Looking at the mechanism, they found that the adsorption of analytes was negatively correlated with their molecular acidity coefficient (p*K*_a_) and positively correlated with their octanol–water distribution coefficient (log *D*) at pH 7, indicating that both the hydrophobic interaction and the electrostatic interaction played a significant role in adsorption. Moreover, by comparing with nonfluorinated fatty acids, the higher adsorption amount for PFAS suggested the existence of fluorophilic interactions.

Using fluorinated cationic surfactant is another way to combine fluorous probes with cationic probes. Du et al. demonstrated selective and strong adsorption of PFOA and PFOS using a fluorinated montmorillonite (F-MT) synthesized via the intercalation of fluorinated cationic surfactants (PFQA) into interlayers of the montmorillonite.^97^ They found that the adsorption amount of PFOA increased with loading concentration of PFQA probes, with a maximum at a loading concentration around 1 mmol/g. They tested the selectivity of F-MT toward PFOS and PFOA against SDBS, pyridine, PHE, and phenol at the same initial concentration of 5 μM, both in individual mode and in mixed mode. The results showed that F-MT had a high selectivity against those organic interferents. The selectivity in this work was mainly attributed to the fluorophilic interactions, while the role of the cations on the intercalated surfactants was not elucidated.

#### 4.3.4 Cavitary Probes

Cavitary probes have been applied extensively into PFAS sensors and sorbents to provide multimode interactions and additional structural confinement. There are many types of cavitary probes, including cyclodextrin-based materials,^139^ MIPs,^61^ porous framework materials like MOFs^64^ and COFs,^50^ synthetic host molecules,^30,110^ as well as the internal structure of minerals.^91,127^ Beta-cyclodextrins (β-CD) and MIPs are the most popular probes used in PFAS sorbents and sensors to date, respectively.

Weiss-Errico et al. used NMR techniques to study the interaction of cyclodextrins (α-CD, β-CD, and γ-CD) with both legacy^140^ and emerging^126^ PFAS molecules. Their results suggested that β-CD formed the strongest complex with PFAS compared to α-CD and γ-CD. These observations were further supported by the molecular sizes of these compounds. The cross-section size for the typical persistence length of fluorocarbon segments of PFAS is 28.3 Å^2^, while the size of the α-CD cavity is 18.9 Å^2^; β-CD is 30.2 Å^2^; and γ-CD is 49.0 Å^2^,^141−143^ which indicates the size of β-CD matches with the fluorinated tail of PFAS. The NMR shifts indicated that PFAS mainly interact with β-CD by inserting the middle section of the fluorinated chain inside the CD cavity. They found that long-chain PFAS could form either a 1:1 or 1:2 complex with β-CD, where the binding affinity of the 1:1 complex is nearly 100 times higher than that of the 1:2 complex for legacy PFAS, while there is no significant difference in binding strength between those two types of complexes for emerging PFAS. They also observed that the strength of the equilibrium constant is influenced by PFAS’ properties like chain length, headgroup, oxygen content, and branched structures. By studying the interaction between branched PFAS with β-CD, they suggested a mechanism of forming hydrogen bonding between the headgroup on PFAS and the primary hydroxyl groups on CD. These results all indicated that β-CD could be a good candidate for selective capture of PFAS.

**Figure 8:**
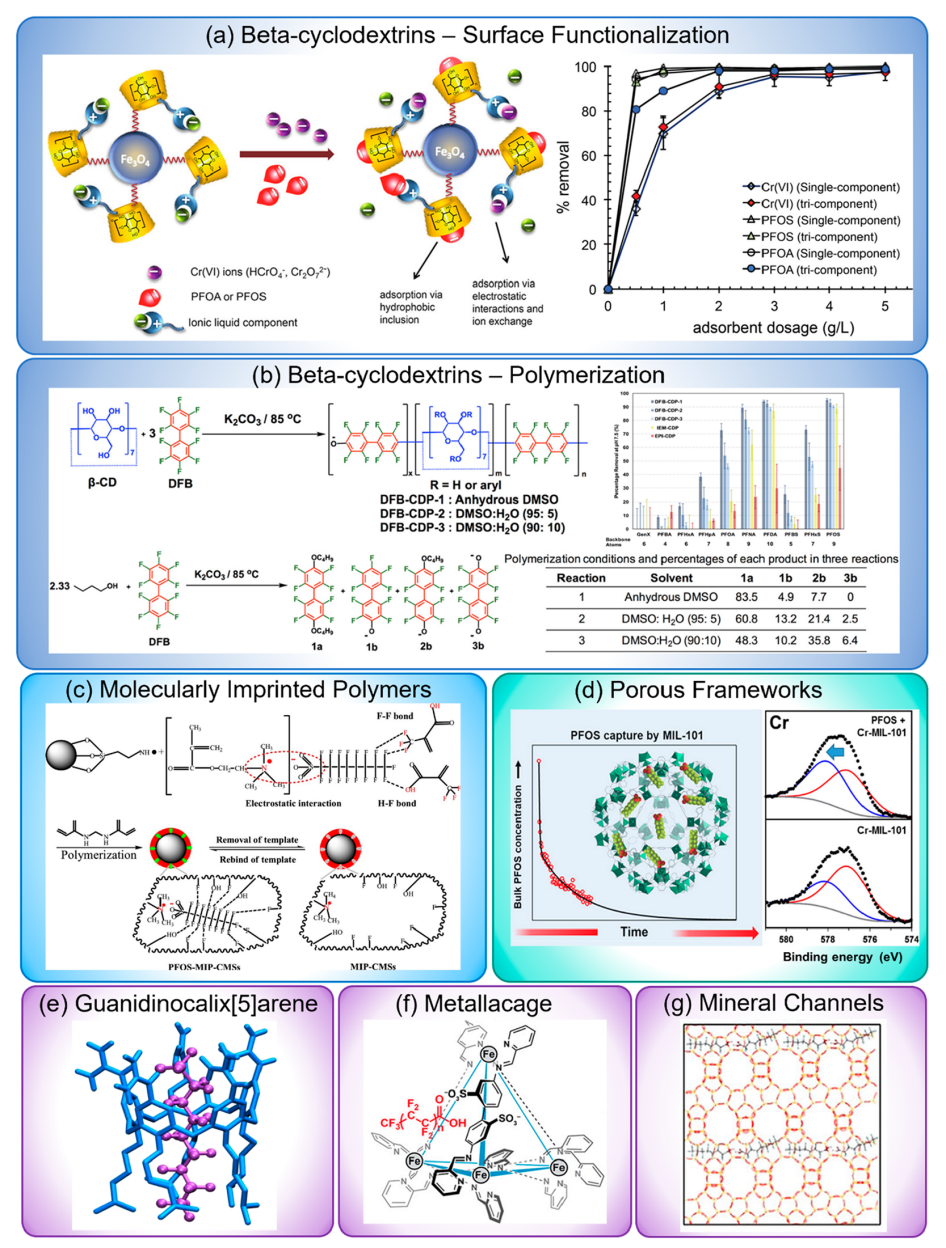
Examples of cavitary probes. (a) A β-CD–ionic liquid (IL) polyurethane-modified magnetic sorbent that achieved simultaneous adsorption of PFOA, PFOS, and Cr (VI) anions. Reprinted with permission from ref (144). Copyright 2017 American Chemical Society. (b) Examination of the influence from the cross-linker chemistry of DFB-CDP on PFAS removal. Reprinted with permission from ref (107). Copyright 2019 American Chemical Society. (c) Carbon microspheres coated with MIP, which was synthesized by copolymerization of fluorinated monomers and quaternary ammonium-containing monomers. Reprinted with permission from ref (79). Copyright 2018 Elsevier. (d) MIL-101 frameworks used to capture PFOS, whose PFOS affinity was characterized by XPS. Reprinted with permission from ref (147). Copyright 2019 American Chemical Society. (e) Guanidinocalix[5]arene as the PFAS-selective probe. Reprinted with permission from ref (30). Copyright 2019 Springer Nature. (f) A self-assembled iron(II) metallacage as a trap for PFAS. Reprinted with permission from ref (125). Copyright 2020 American Chemical Society. (g) Channels inside all-silica zeolite-β capable of capturing PFOA, as calculated by DFT. Reprinted from with permission ref (90). Copyright 2020 Wiley.

To optimize the capture performance of β-CD, they are often combined with other functional materials in PFAS sorbents. Badruddoza et al.^144^ designed and synthesized a β-CD–ionic liquid (IL) polyurethane-modified magnetic sorbent to achieve simultaneous adsorption of PFOA, PFOS, and Cr(VI) anions (Figure 8a). They prepared this sorbent by first transforming the hydroxyl groups of β-CD to cationic imidazolium groups to form β-cyclodextrin ionic liquid (β-CD-IL). Then they attached β-CD-IL onto magnetic nanoparticle (MNP) surfaces by using hexamethylene diisocyanate (HMDI) as the linker, resulting in β-CD-IL polyurethane-functionalized Fe_3_O_4_. They tested the removal of PFOA, PFOS, and Cr(VI) individually and simultaneously and found the removal efficiencies of these analytes were not affected by each other except for PFOA at low sorbent dosage (<2 g/L). They explained that the dual binding sites rendered by β-CD moieties and IL components provided host–guest inclusion and ion-exchange capabilities, resulting in the novel adsorption performance. Xiao et al. synthesized a β-CD polymer network (DFB-CDP) by using decafluorobiphenyl (DFB) as the cross-linker.^40^ By changing the DFB/β-CD ratio, they found the optimal adsorption happened at a ratio of 3:1. Further increasing the DFB (the fluorinated cross-linker) concentration would reduce the removal percentage by rendering too much steric hindrance in the densely cross-linked network. Using DFB-CDP, they were able to reduce PFOA concentrations from 1 μg/L to <10 ng/L, and the adsorption was unaffected by the presence of humic acid. In a following study, Xiao et al. examined the influence of the cross-linker chemistry by polymerizing β-CD and DFB with the same molar feed ratio 1:3 in different solvents (Figure 8b).^107^ Three DFB-CDPs were synthesized with similar cross-linking density but different phenolate (having negative charges) concentrations. The results showed that the sorbents with the lowest phenolate concentration exhibited the highest affinity for 10 PFAS in buffered solutions. Their findings indicated that negative charges around β-CD hindered the capture efficiency of anionic PFAS. Further modification of the cross-linker for β-CD polymers by using amine-containing cross-linkers reduced from tetrafluoroterephthalonitrile (TFN) was explored by Klemes et al.,^139^ and using tris(2-aminoethyl)amine (TREN)-based tripodal cross-linkers was reported by Yang et al.^145^ Both materials exhibited excellent removal percentage, indicating a beneficial combination of electrostatic attraction and host–guest interaction for PFAS adsorption. A similar strategy of cross-linking host molecules by a functional cross-linker was adopted by Shetty et al. by polymerizing calixarene macrocycles with four linkers differing in number of aromatic rings and fluorine content (Figure 4h).^110^ The results showed that polymers with fluorinated linkers have a higher adsorption capacity than those with nonfluorinated linkers, indicating the presence of C–F···F–C interaction. However, their Monte Carlo (MC) simulations implied that hydrogen bonding was the main interaction between the sorbents and their environment, while the effect of C–F···F–C interaction is marginal. Instead, the role of fluorination was to create new binding sites on the linkers for the adsorption of PFOA, which no longer needed to compete with water.

MIPs have been the dominant choice among selective probes used in PFAS sensors.^44,60,61,65,67−69,120,121^ PFAS-specific MIPs are synthesized by copolymerizing functional monomers and cross-linkers in the presence of PFAS templates. After the removal of templates, the remaining cavities inside MIPs match with template molecules in size, shape, and functional groups, which enables MIPs to selectively recognize template molecules. In sensors, MIPs are often used as a door (MIP film) with a specific lock (templated cavity) toward a certain key molecule (PFAS template). The “turn on/off” response of the sensor induced by decreasing/increasing the number of PFAS molecules could be utilized to detect PFAS concentrations. Polymeric materials that have been used to imprint molecules in PFAS sensors and sorbents include, but are not limited to, poly(*o*-phenylenediamine) (poly(*o*-PD)),^61^ polyacrylamide,^66^ polypyrrole,^69^ silica-based materials,^121^ and chitosan-based materials.^44^ Guo et al. incorporated both fluorinated probes and cationic probes into MIPs by copolymerizing 2-(trifluoromethyl) acrylic acid (TFMA) and methacryloyloxyethyl trimethylammonium chloride (DMC) at a 1:1 molar ratio (Figure 8c).^79^ Most MIP-based PFAS sensors and sorbents exhibited some selectivity even within the PFAS family,^66^ but there remains room for improvement. Several studies indicate that the templated cavities in MIPs could be occupied by molecules with smaller sizes but similar functional groups, which is a challenge to overcome in designing higher selectivity.^60,146^

Porous framework materials including MOFs and COFs are strong candidates for selective PFAS capture. Barpaga et al. examined the potential of using Cr-MIL-101 and Fe-MIL-101 for PFOS capture by using various characterization tools to study the interactions between PFOS and these two MOF candidates (Figure 8d).^147^ They found that Cr-MIL-101 had faster capture kinetics as well as higher binding affinity toward PFOS than Fe-MIL-101. This might be due to the strong interaction between sulfur atoms of the headgroup of PFOS and the Cr center of MOF, as revealed by XPS analysis. Cr-MIL-101 was later incorporated into a microfluidic impedance sensor platform in another study and achieved ultrasensitive detection of PFOS with a LOD of 0.5 ng/L.^64^ Two studies either introducing fluorinated moieties into UiO-66^132^ or adding amine-containing moieties into MIL-101(Cr)^111^ both showed that the functional probe-modified MOFs had higher adsorption performance than the pristine MOF structure, indicating the benefits of combining multiple interactions. Similarly, embedding COFs into sensors could render a high sensitivity.^50^ Amine^112^ or fluorous^148^ functionalities could be added to COFs to improve the PFAS capture.

Some other synthetic cavitary molecules have been applied for PFAS encapsulation as well. Zheng et al. synthesized a guanidinocalix[5]arene-based probe and studied its interaction with PFAS via computational methods complemented by NMR (Figure 8e).^30^ They explained that this guanidinocalix[5]arene could complex with PFOS and PFOA by salt–bridge interactions and size complementarity. They calculated the binding constant for guanidinocalix[5]arene toward PFOS and found it to be higher than other reported host molecules for PFOS, including β-CD. By applying an indicator displacement assay (IDA) (Figure 3a), they were able to use this probe to achieve selective fluorometric detection of PFOA and PFOS. The selectivity was tested against CTAB, octanesulfonic acid, octanoic acid, perfluorohexane, NaCl, Na_2_SO_4_, KCl, MgCl_2_, and solutes in wastewater, with the sensor showing little response to those interferents. By coassembly with magnetic nanoparticles, guanidinocalix[5]arene could be used as a PFAS sorbent and could be simply separated from the sample solution by applying an external magnetic field. In another study, a self-assembled iron(II) metallacage was synthesized and examined as a trap for PFAS (Figure 8f).^125^ It was found that this cage could remove more PFAS with longer chain lengths (C ≥ 6) and smaller headgroups compared to those with smaller chains and containing sulfonamides. Future work is needed to better match the internal cavity size of the cage with the size of specific PFAS molecules to promote host–guest interactions between PFAS and the cage. In some cases, the interlayer space and the porous structures in minerals could also act as a host to accommodate PFAS molecules (Figure 8g).^89,90^

## 5 Conclusions and Future Perspectives

Facing the grand challenges of water contamination caused by PFAS, efficient and effective methods for the detection and removal of PFAS in aquatic environments are of vital importance. In this review, we summarized the current sensing technologies for PFAS according to their sensing mechanism and introduced sorbents that have been tested for PFAS removal based on a variety of material classes. Special attention is drawn to the selectivity of the existing PFAS sensors and sorbents, including the role of common interferents. Possible interacting mechanisms underlying selectivity were analyzed cooperatively with the introduction of selective probes for PFAS. Fluorinated probes, cationic probes, and cavitary probes are the three most common functional probes used to date. The functions and interplay of those probes and strategies to incorporate those probes into materials and devices were discussed in detail. Such probe structures and material design strategies provide guidance to achieve even more selective detection and adsorption of PFAS in future systems.

Insufficient selectivity is the common issue underlying all current PFAS sensing and adsorption technologies. While there are many PFAS studies in the literature, there are a limited number of reports on PFAS sensors and sorbents that have evaluated selectivity. For those that have explored selectivity, the experimental conditions varied substantially, making it difficult to systematically examine and compare the selectivity among existing sensors and sorbents. Moreover, some selectivity tests were conducted under unrealistic conditions by applying, for example, unrealistically high PFAS concentrations. The selections of interferents for PFAS in current research would benefit from additional justification and ideally would reflect realistic environments. Future research would benefit from rational and realistic design of selectivity experiments. Elucidation of interacting mechanisms without the assistance of computational simulation can be challenging. Simulating without the presence of water, for example, might weaken the conclusions. To address the selectivity issue, a more thorough understanding of PFAS-involving interactions is needed. Current interaction mechanisms are proposed mainly according to empirical observations in adsorption experiments. Such adsorption mechanisms are not exactly the same as the molecular interactions between PFAS and other functional probes, as adsorption is a complex process influenced by sorbent properties, PFAS characteristics, solution chemistry, as well as the experimental conditions and methods. It is extremely challenging to decouple all those factors and extract molecular interactions of PFAS independently. Therefore, fundamental studies focusing on simpler material or molecular systems to probe the PFAS interactions are suggested in order to understand each interaction more thoroughly. It would be helpful if numeric parameters could be proposed to compare the interaction strength quantitatively. This approach would be beneficial for future probe design to realize higher selectivity toward PFAS, especially when facing the strict requirement for PFAS sensors to distinguish PFAS molecules within their large family. Finally, gaining a deeper understanding of PFAS interactions and designing more selective probes will not only improve the selectivity of sensors and sorbents but also contribute to other parameters like the sensitivity for sensors and the adsorption capacity and kinetics for sorbents. However, high selectivity is not always a good thing, as strong binding will make the desorption of PFAS difficult, which is not favorable for the regeneration of PFAS sorbents. Although many studies have demonstrated the reusability of highly selective PFAS sorbents, most of them used organic solvents^40,108,135^ like methanol, which are hazardous, and thus are not recommended for water treatment applications. Less harmful solvents like brine, which are commonly employed in industry, may be ineffective for the regeneration of PFAS-specific sorbents.^116,149^ The trade-off between the high selectivity that is beneficial for adsorption performance and cost-effectiveness and the reusability which promotes sustainability and environmentally friendliness is an important point to be considered when designing PFAS-capturing materials. A subtle molecular design of PFAS-selective probes where the binding strength could be tuned to a moderate or optimized level would be helpful to reach a good balance between different properties of PFAS sorbents as well as sensors. All these efforts will eventually promote the development of PFAS monitoring and remediation methods and facilitate progress to mitigate the ongoing PFAS exposure.
